# Dynamic regulation of genome-wide pre-mRNA splicing and stress tolerance by the Sm-like protein LSm5 in *Arabidopsis*

**DOI:** 10.1186/gb-2014-15-1-r1

**Published:** 2014-01-07

**Authors:** Peng Cui, Shoudong Zhang, Feng Ding, Shahjahan Ali, Liming Xiong

**Affiliations:** 1 Biological and Environmental Sciences and Engineering Division, King Abdullah University of Science and Technology (KAUST), Thuwal 23955-6900, Saudi Arabia

## Abstract

**Background:**

Sm-like proteins are highly conserved proteins that form the core of the U6 ribonucleoprotein and function in several mRNA metabolism processes, including pre-mRNA splicing. Despite their wide occurrence in all eukaryotes, little is known about the roles of Sm-like proteins in the regulation of splicing.

**Results:**

Here, through comprehensive transcriptome analyses, we demonstrate that depletion of the *Arabidopsis* supersensitive to abscisic acid and drought 1 gene (*SAD1*), which encodes Sm-like protein 5 (LSm5), promotes an inaccurate selection of splice sites that leads to a genome-wide increase in alternative splicing. In contrast, overexpression of *SAD1* strengthens the precision of splice-site recognition and globally inhibits alternative splicing. Further, *SAD1* modulates the splicing of stress-responsive genes, particularly under salt-stress conditions. Finally, we find that overexpression of *SAD1* in *Arabidopsis* improves salt tolerance in transgenic plants, which correlates with an increase in splicing accuracy and efficiency for stress-responsive genes.

**Conclusions:**

We conclude that *SAD1* dynamically controls splicing efficiency and splice-site recognition in *Arabidopsis*, and propose that this may contribute to *SAD1*-mediated stress tolerance through the metabolism of transcripts expressed from stress-responsive genes. Our study not only provides novel insights into the function of Sm-like proteins in splicing, but also uncovers new means to improve splicing efficiency and to enhance stress tolerance in a higher eukaryote.

## Background

Immediately following transcription, many eukaryotic precursor messenger RNAs (pre-mRNA) are subjected to a series of modifications that are essential for the maturation, nuclear export and subsequent translation of these transcripts. One such modification, the removal (splicing) of non-protein-coding sequences from pre-mRNA, is an important step in gene regulation that also contributes to increased protein diversity from a limited number of genes. The precision and efficiency of splicing are critical for gene function [1]. A non-precision splicing process would generate aberrant or non-functional mRNAs that are not only wasteful but can also lead to the production of unwanted or harmful proteins that may perturb normal cellular processes. Moreover, incorrectly spliced transcripts might also have a profound impact on other processes, including mRNA transcription, turnover, transport and translation. Accumulating evidence indicates that poor efficiency or defects in splicing can lead to diseases in humans [2,3] and increase sensitivity to abiotic or biotic stresses in plants [4-6]. Although many molecular processes related to splicing have been well characterized, we still face a major challenge in understanding how precision and efficiency in splicing are regulated and how we could harness these regulations to enhance cellular functions.

Sm-like proteins (LSms) are a highly conserved family of proteins in eukaryotes both in terms of sequence and functions. LSms typically exist as heptameric complexes and play roles in multiple aspects of RNA metabolism [7-9]. The heptameric LSm1-7 cytoplasmic complex is located in discrete cytoplasmic structures called P-bodies, which are conserved in all eukaryotes and are thought to be involved in decapping and 5′ to 3′ RNA degradation [10,11]. The LSm2-8 heptameric complex is located in the nucleus. This complex directly binds and stabilizes the 3′-terminal poly(U) tract of U6 small nuclear RNA, forms the core of the U6 small nuclear ribonucleoproteins (RNPs) and functions in pre-mRNA splicing [12,13]. The *Arabidopsis* supersensitive to abscisic acid (ABA) and drought 1 (*SAD1*) gene locus encodes the LSm5 protein and was identified in a genetic screen for components that regulate the expression of stress-responsive genes in our previous work [14]. SAD1 directly interacts with two other subunits, LSm6 and LSm7, and is a component of the LSm2-8 nuclear complex [15]. Dysfunction of SAD1 increases the plant’s sensitivity to salt stress and to the stress hormone ABA in seed germination and root growth; moreover, *sad1* mutants are defective in the positive feedback regulation of ABA biosynthesis genes by ABA and are impaired in drought stress induction of ABA biosynthesis, although the detailed molecular bases for these defects have not been identified. Recent studies suggested that the depletion of SAD1 and the other LSm protein (LSm8) reduced the stability of U6 RNPs and resulted in defects in pre-mRNA splicing that lead to intron retention in *Arabidopsis *[15,16]. However, it is still unclear if the depletion of SAD1 or other LSm proteins has any effect on the selection of splice sites and alternative splicing (AS), and whether overexpression of these LSm proteins could affect splicing efficiency or accuracy.

To investigate possible regulatory roles of SAD1 protein in pre-mRNA splicing, we performed RNA sequencing (RNA-seq) of the wild-type *Arabidopsis* (C24 ecotype), the *sad1* mutant and the *SAD1*-overexpressing plants (SAD1-OE). We found that SAD1 could dynamically control splicing efficiency and splice-site recognition and selection in *Arabidopsis*. Additionally, we discovered that SAD1 is required for regulation of splicing efficiency of many stress-responsive genes under stress conditions. Whereas there are increased splicing defects in *sad1* mutants under salt-stress conditions, overexpression of *SAD1* increases the splicing efficiency of stress-related genes. SAD1-OE plants are also more salt-tolerant than wild-type plants. Our work not only provides novel insights into the regulatory role of SAD1 and LSm proteins in splicing, but also suggests a new way to improve splicing efficiency and to optimize cellular functions and generate stress-resistant plants.

## Results

### RNA sequencing of wild-type, *sad1* mutant and *SAD1*-overexpressing plants

The *Arabidopsis sad1* mutant was isolated in our previous genetic screen for components that regulate the expression of stress-responsive genes [14]. The *sad1* mutant was also more sensitive to stress and ABA inhibition of seed germination and seedling growth [14]. Since loss-of-function mutations in any single-copied core LSm genes are expected to be lethal, the recovery of this point-mutation *sad1* mutant provided an invaluable opportunity to study the functions of this important group of proteins. To explore the role of SAD1 in gene expression and stress tolerance, we generated transgenic *Arabidopsis* plants over-expressing the wild-type *SAD1* cDNA (under control of the cauliflower mosaic virus *35S* promoter) both in the wild type (ecotype C24) and in the *sad1* mutant background (SAD1-OE, see Methods). Although the transgenic plants in both backgrounds had similar physiological and molecular phenotypes, here we mainly focus on SAD1-OE in the *sad1* mutant background (referred to as SAD1-OE hereafter).

**Figure 1:**
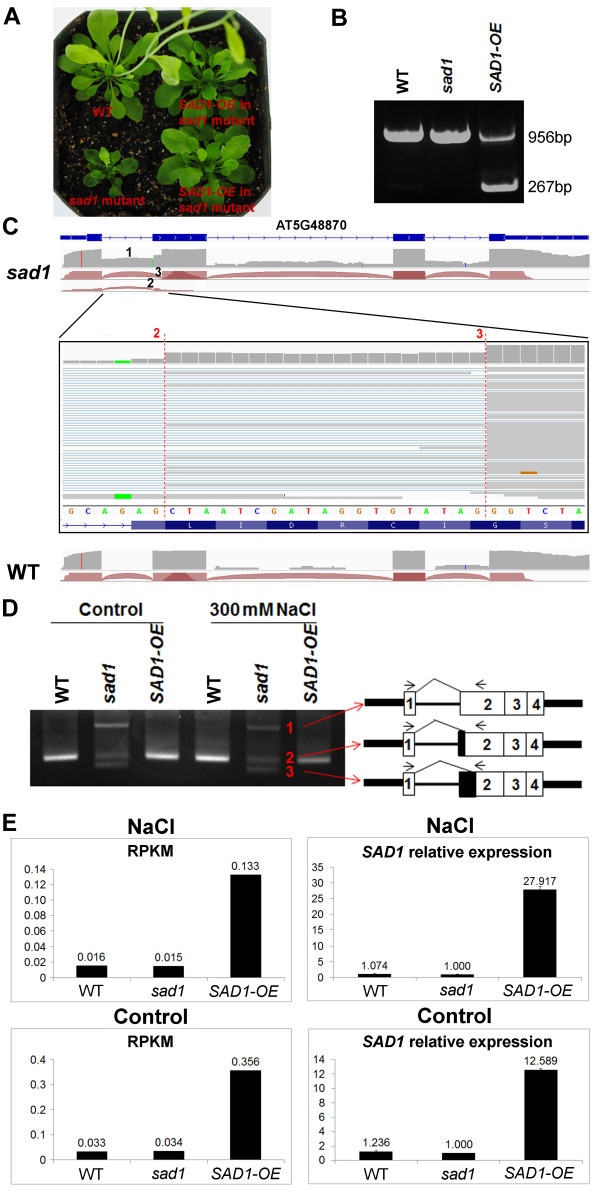
**Generation of the *SAD1*-overexpressing transgenic plants (SAD1-OE) and the splicing variants of *SAD1 *in the wild type, *sad1 *and SAD1-OE. (A)** Morphology of the wild type, *sad1* and SAD1-OE seedlings in soil. **(B)** Genotype analysis of plants shown in **(A)**. The upper and lower bands of PCR products represent the endogenous *SAD1* gene and the transgenic cDNA, respectively. **(C)** RNA-seq reads were visualized by the Integrative Genomics Viewer (IGV) browser across the *SAD1* gene. Exon-intron structure was given at the bottom of each panel. The arcs generated by IGV browser indicate splice junction reads that support the splice junctions. The grey peaks indicate RNA-seq read density across the gene. The upper panel depicts the mutation of *sad1* that changed the wild-type invariant dinucleotide AG to AA at the 3′ splicing acceptor recognition site of the first intron. The middle panel shows transcripts with two aberrant 3′ splice sites (3′SSs) that respectively occurred at the 20 bp (enlarged and marked by 3) and 2 bp (enlarged and marked by 2) downstream of the mutated splice site and transcripts with the retention of the first intron (marked by 1) in *sad1*. Also shown are *SAD1* transcripts in the wild type where they were normally spliced. **(D)** Three variants of *SAD1* transcripts discovered in *sad1* by RNA-seq were validated by RT-PCR using junction-flanking primers. The three bands in the *sad1* mutant from top to bottom represent transcripts with the first intron retained, the first aberrant 3′SSs and the second aberrant 3′SSs, respectively. Note in the wild-type and SAD1-OE plants, only one wild-type *SAD1* band was detected. **(E)***SAD1* expression levels were shown using the reads per kilobase per million value and quantitative RT-PCR. bp, base pairs; RPKM, reads per kilobase per million; *sad1*, *sad1* mutant; SAD1-OE, plants over-expressing wild-type *SAD1* in the *sad1* mutant background; WT, wild type.

As shown in Figure 1A, overexpressing wild-type *SAD1* rescued the small stature phenotype of the *sad1* mutant, demonstrating that the phenotypic defects of the *sad1* mutant were caused by the loss of the wild-type SAD1 protein. We genotyped these seedlings using primers that span the whole gene body. The PCR products in the SAD1-OE plant had two bands, representing the original *SAD1* gene and the transferred cDNA, respectively (Figure 1B).

We next performed RNA-seq using the Illumina HiSeq platform (Illumina Inc., San Diego, CA, USA) on two-week-old seedlings of C24 (wild type), *sad1* and SAD1-OE. These seedlings were subjected to two treatments: control (H_2_O) and salt stress (300 mM NaCl, 3 h). The salt-stress treatment was based on our previous observations that stress-responsive genes were most obviously activated and that the mutant *sad1* showed strong molecular phenotypes under these conditions [14,17]. Based on six cDNA libraries (C24-control, *sad1*-control, SAD1-OE*-*control, C24-NaCl, *sad1*-NaCl and SAD1-OE*-*NaCl), we generated a total of 164 million reads (101 bp in length, except for the SAD1-OE control, whose reads were 85 bp in length), about 90% of which could be uniquely aligned to the TAIR10 reference genome sequence (version TAIR10; [18]) (Additional file 1). Comparison of the mapped reads to the gene model (version TAIR10) revealed that approximately 95% of the reads mapped to the exonic regions, whereas only about 3% mapped to intergenic regions (Additional file 2), which were consistent with the *Arabidopsis* gene annotation. Plotting the coverage of reads along each transcript exhibited a uniform distribution with no obvious 3′/5′ bias, which reflects the high quality of the cDNA libraries (Additional file 3). Furthermore, assessing the sequencing saturation demonstrated that as more reads were obtained, the number of new discovered genes plateaued (Additional file 4). This suggests that extensive coverage was achieved, which can also be seen when the read coverage was plotted by chromosome, demonstrating extensive transcriptional activity in the genome (Additional file 5).

We previously identified the *sad1* mutation as a G-to-A change 34 bp from the putative translation start site and predicted that the mutation would change a glutamic acid (E) residue to lysine (K). In the RNA-seq data, the mutation of *sad1* at the genomic position 19,813,556 of chromosome 5 was confirmed. However, it turned out that the mutation occurred at the 3′ splicing acceptor recognition site of the first intron, changing the invariant AG dinucleotide to AA. Consequently, all of *sad1* mRNAs were aberrantly spliced in the mutants, as visualized with the Integrative Genomics Viewer (IGV) browser [19,20] (Figure 1C). We identified three main mutant transcripts in *sad1*: two with obvious aberrant 3′ splice sites (3′SSs) that respectively occurred 2 bp and 20 bp downstream of the mutated splice site; and one with the retention of the first intron (Figure 1C). All of these transcripts were validated by RT-PCR using primers that span the alternative 3′SSs, in which the corresponding events were detected in the *sad1* mutant, but not in C24 (Figure 1D). Sequence analysis suggested that the transcript with aberrant 3′SSs that occurred 20 bp downstream of the mutated splice site did not alter the coding frame. It was predicted to produce one novel protein with the deletion of seven amino acids compared to the normal SAD1 protein. It seems that this mutant protein could provide some of the wild-type’s functions such that the *sad1* mutation was not lethal. By contrast, the other alternative 3′SS and the intron retention led to a coding-frame shift that would generate a premature stop codon and thus would lead to truncated proteins. In the SAD1-OE plant, all these aberrantly spliced forms could be found, albeit at much lower levels than in *sad1*. However, normal *SAD1* mRNA was overexpressed, with the transcript level more than 10-times higher than in C24, which was validated by quantitative RT-PCR (Figure 1E).

### Identification of alternative splicing events in C24, *sad1* and SAD1-OE plants

To determine if there were any changes in pre-mRNA splicing upon the depletion or overexpression of *SAD1*, we first developed a pipeline to identify all AS events in C24, *sad1* and SAD1-OE. The pipeline involved three steps: prediction of splice junctions, filtering of the false positive junctions and annotation of AS events. We randomly sampled 20 million uniquely mapped reads (estimated average approximately 57-times coverage on all the expressed transcripts) from each RNA-seq library for the identification or comparison of AS, respectively. This method ensured that the comparison of AS events would be performed at the same level.

To predict splice junctions, we mapped the RNA-seq reads onto the *Arabidopsis* genome using the software TopHat, which was designed to identify exon-exon splice junctions [21]. After the alignment, we identified 732,808 junctions from the six RNA-seq libraries. Comparison of these junctions to the gene annotation (TAIR10) revealed that about 83% of total junctions had previously been annotated, and the remaining 17% were assigned as novel junctions (Additional file 6A). However, when trying to characterize these novel and annotated junctions, we found that there was a large number of novel junctions that had short overhangs (that is, fewer than 20 bp) with the corresponding exons, while most of the annotated junctions had large overhangs, with the enrichment at approximately 90 bp (Additional file 6B). Moreover, the novel junctions had relatively low coverage compared with the annotated junctions (Additional file 6C). In general, the junctions with short overhangs and lower coverage were considered as false positives, which are often caused by non-specific or error alignment. Therefore, to distinguish between true splice junctions and false positives, we assessed the criteria based on simulated data of a set of randomly constituted junctions. To do this, we first generated a set of 80,000 splice junctions in which annotated exons from different chromosomes were randomly selected and spliced together *in silico*. We also constructed 119,618 annotated junctions from the gene annotation. Since the length of our sequencing reads was 101/85 bp, the splice junction sequences were determined to be 180/148 bp long (90/74 nucleotides on either side of the splice junction) to ensure an 11 bp overhang of the read mapping from one side of the junction onto the other. Alignments to the random splice junctions were considered to be false positives, because such junctions are thought to rarely exist when compared to annotated junctions. The alignment of the raw RNA-seq reads to the random junctions revealed that 99.90% of false positive junctions had an overhang size of fewer than 20 bp (Additional file 7A). In sharp contrast, the alignment to the annotated junctions indicated that most (98.60%) annotated junctions had larger overhang sizes. In addition, we estimated that 56.90% of false positive junctions had only one read spanning the junction, whereas the annotated junctions had higher read coverage (Additional file 7B). To minimize the false positive rate, we required that the overhang size must be more than 20 bp and that there be at least two reads spanning the junctions. Using these criteria, we filtered out almost all the false positive junctions (Additional file 7C). Finally, we obtained a junction data set of 52,599 confident novel junctions from the six RNA-seq libraries. Based on these junctions, we identified all the AS events including cassette exons, alternative 5′SSs, alternative 3′SSs, mutually exclusive exons, coordinate cassette exons, alternative first exons and alternative last exons (Additional file 8).

### Depletion of SAD1 activates alternative splicing

**Figure 2:**
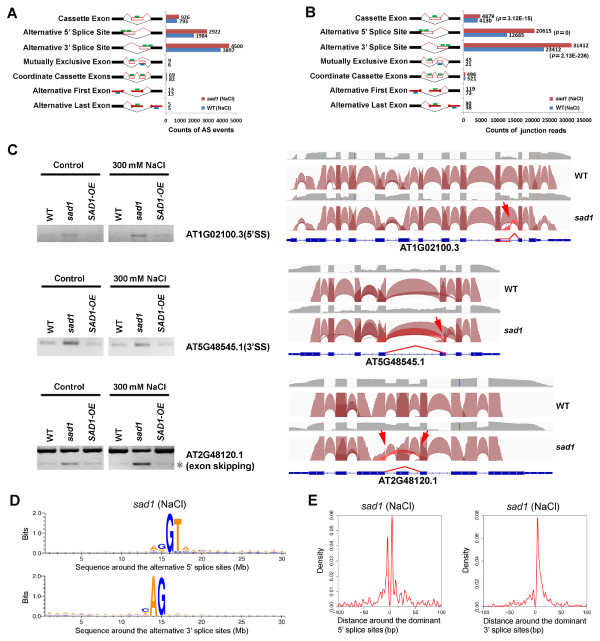
**Comparison of global alternative splicing between the wild type and the *sad1 *mutant. (A)** The counts of each type of AS events in the wild type and *sad1*. The green/blue bars represent forward and reverse sequencing reads. **(B)** The total counts of the splice junction reads from each type of AS in the wild type and *sad1*. The *P* values were calculated by Fisher’s exact test comparing the junction read counts and the uniquely mapped reads between the wild type and *sad1*. **(C)** Three representative AS events validated by RT-PCR and visualized by IGV browser. For the validation of alternative 5′SSs and 3′SSs, there was only one band that represented the alternative-splice isoform, which was obviously detected in *sad1* mutants, but not in the wild type and SAD1-OE. For exon-skipping events, the grey asterisk (*) to the right side denotes the alternative splice form. For the IGV visualization, exon-intron structure of each gene was given at the bottom of each panel. The arcs generated by IGV browser indicate splice junction reads that support the junctions. The grey peaks indicate RNA-seq read-density across the gene. The upper, middle and lower panels show the indicated genes with alternative 5′SSs, alternative 3′SSs and exon-skipping, respectively. These events were marked by red arrows and highlighted by red arcs. **(D)** The sequences around the alternative 5′SSs and 3′SSs that were over-represented in the mutant are shown by Weblogo. **(E)** Distribution of activated alternative 5′SSs and 3′SSs around the dominant ones. These alternative 5′SSs and 3′SSs were enriched in the downstream or upstream 10 bp region of the dominant 5′SSs and 3′SSs (position 0 on the *x*- axis), respectively. AS, alternative splice; *sad1*, *sad1* mutant; SAD1-OE, plants over-expressing wild-type *SAD1* in the *sad1* mutant background; WT, wild type.

We first compared the difference in AS between C24 and the *sad1* mutant. By comparing the number of AS events, we found that the alternative 5′SSs and exon-skipping events were consistently promoted in the control and NaCl-treated mutants (Figure 2A; Additional file 9A). Furthermore, the number of splice junction reads from alternative 5′SSs and exon-skipping events in the mutant was significantly higher than that in the wild type (Fisher’s exact test, *P* <0.001) (Figure 2B; Additional file 9B). Using Fisher’s exact test on the junction read counts and the corresponding exon read counts between the wild type and the mutant, we identified 478 alternative 5′SSs and 138 exon-skipping events from 550 genes that were significantly over-represented in the control or NaCl-treated mutants; by contrast, we identified only 133 alternative 5′SSs and 41 exon-skipping events from 171 genes that were over-represented in the corresponding wild type (Additional files 10, 11, 12 and 13). These results indicated that SAD1 depletion increased alternative 5′SSs and exon-skipping events. In addition, the alternative 3′SSs showed significant increases in the NaCl-treated mutant. We identified 319 alternative 3′SSs that were over-represented in the mutant; by contrast, 142 were over-represented in the wild type (Additional files 14, 15). This result suggests that SAD1-depletion could also promote alternative 3′SSs under salt-stress conditions.

Twenty-two selected events were further validated by RT-PCR using the splicing-site-flanking primers, in which the corresponding AS events were detected in *sad1* mutants, but were weakly or not presented in C24 (Figure 2C and Additional file 16). Figure 2C highlights three representative examples visualized by the IGV junction browser and validated by RT-PCR. The *SBI1* (AT1G02100) gene had alternative 5′SSs in the 10th intron in *sad1*, but not in C24, an observation validated by RT-PCR using the forward primer that covered the splice junction and the reverse one that was located at the 11th exon. One can see that the corresponding isoform was detected in the *sad1* mutant, but was not present in C24 (Figure 2C). The *HINT3* (AT5G48545) gene had alternative 3′SSs in the fifth exon in the mutant *sad1*, which was validated by RT-PCR using a forward primer in the first exon and a reverse primer that covered the splice junction (Figure 2C). The gene *PAC* (AT2G48120) exhibited exon-skipping between the third and fifth exons, which was validated by RT-PCR using primers at the third and sixth exon, which meant that two different products were amplified, representing exon inclusion and skipping isoforms, respectively (Figure 2C).

Sequence analysis of these over-represented alternative 5′SSs and alternative 3′SSs (in the NaCl-treated *sad1* mutant) revealed that these activated splice sites were still associated with GU and AG dinucleotides (Figure 2D; Additional file 17A), suggesting that the depletion of SAD1 did not change the accuracy of the sequence recognition of the splicing sites. When investigating the distribution of these activated splice sites, we found that alternative 5′SSs and 3′SSs were enriched in the downstream or upstream approximately 10 bp region of the dominant 5′SSs and 3′SSs, respectively (Figure 2E; Additional file 17B). This indicates that the depletion of SAD1 leads to the activation of the 5′SSs and 3′SSs proximal to the respective dominant ones. These results suggest that SAD1, as a component of U6 RNPs, may play a regulatory role in the selection of splice sites.

Interestingly, exon-skipping events also increased in *sad1* mutants. When correlating each exon-skipping event with alternative 5′SSs and 3′SSs, we found that about 20% of the skipped exons simultaneously had alternative 5′SSs or 3′SSs in the mutants. This chance of occurrence was significantly higher than that expected for random sampling of all annotated exons (the probability of random occurrence was 0.02%, Fisher’s exact test, *P* <0.001). This result suggests a coordinated occurrence of exon-skipping and alternative splice site selection. Therefore, we considered that SAD1-depletion could simultaneously activate multiple alternative 5′SSs or 3′SSs that include not only the proximal ones, but also the distal ones, including those located at the next exons, albeit to a lesser extent. Nonetheless, the possibility that SAD1, probably as a splicing factor, may directly regulate exon-skipping *in vivo* could not be ruled out.

### SAD1 depletion results in widespread intron retention

**Figure 3:**
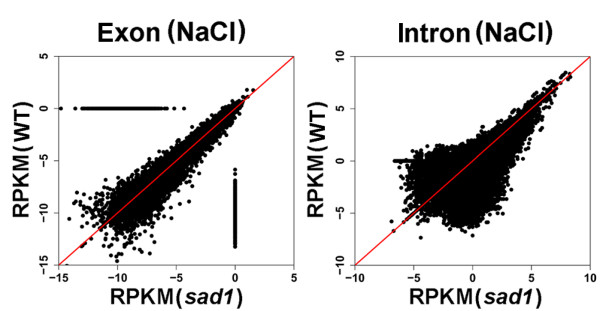
**Comparison of intron retention between the wild type and *sad1*.** The RPKM values for the exons and introns were plotted between the wild type and *sad1*. The expression of introns in the *sad1* mutant shows a global up-regulation, but not that of exons. RPKM, reads per kilobase per million; *sad1*, *sad1* mutant; WT, wild type.

Based on DNA chip and RT-PCR analyses, very recent studies have suggested that the depletion of SAD1 and other LSm proteins can result in defects in intron removal [15,16]. Nonetheless, genome-wide analyses at the single nucleotide level of splicing defects in these mutants are not available. Based on our RNA-seq data, we plotted the expression intensity of introns and exons between the wild-type C24 and *sad1* mutants (Figure 3; Additional file 18). Figure 3 clearly shows a global up-regulation of intron expression in the mutants, but this was not seen for exon expression, suggesting widespread intron retention in the mutant. Ten selected events were further validated by RT-PCR using the intron-flanking primers, in which the corresponding intron retention events were detected in *sad1* mutants, but were weakly or not presented in C24 (Additional file 19). Using Fisher’s exact test, we compared the counts of intron reads and the corresponding counts of exon reads between the wild type and mutants. We identified 4,610 introns from 2,737 genes that were significantly retained in the control or NaCl-treated mutants (*P* <0.001) (Additional file 20). By contrast, only 23 introns from 20 genes were significantly retained in the corresponding wild-type plants (Additional file 21). This result further demonstrated that SAD1 depletion results in widespread intron retention.

We next investigated if there is any influence of the splicing defects on the expression of the affected genes. Sequence analysis suggested that all of these intron retention events would generate premature stop codons in the intron-retained transcripts and, if translated, would produce truncated proteins. Although it is possible that some individual truncated proteins might still be functional, for our sequence analyses, we assumed that these intron-retained transcripts do not generate functional proteins. Through calculating the proportions of the intron-retained transcripts to the total transcripts for each gene with intron-retention in the mutant, we estimated that on average around 15% of total transcripts were with intron retention (Additional file 22). Moreover, when plotting the expression levels of the total and the functional transcripts (without intron) for each intron-retained gene between the wild type C24 and *sad1* mutants (Additional files 23 and 24), we found that the expression levels of the total transcripts did not obviously change between C24 and *sad1*, but the functional transcripts tended to be down-regulated in the mutant. These results indicate that the splicing defects are associated with a global reduction of functional mRNAs, which might negatively affect the functions of these affected genes.

### Genes with aberrant splicing in *sad1* are closely related to stress response and are activated by stress

**Figure 4:**
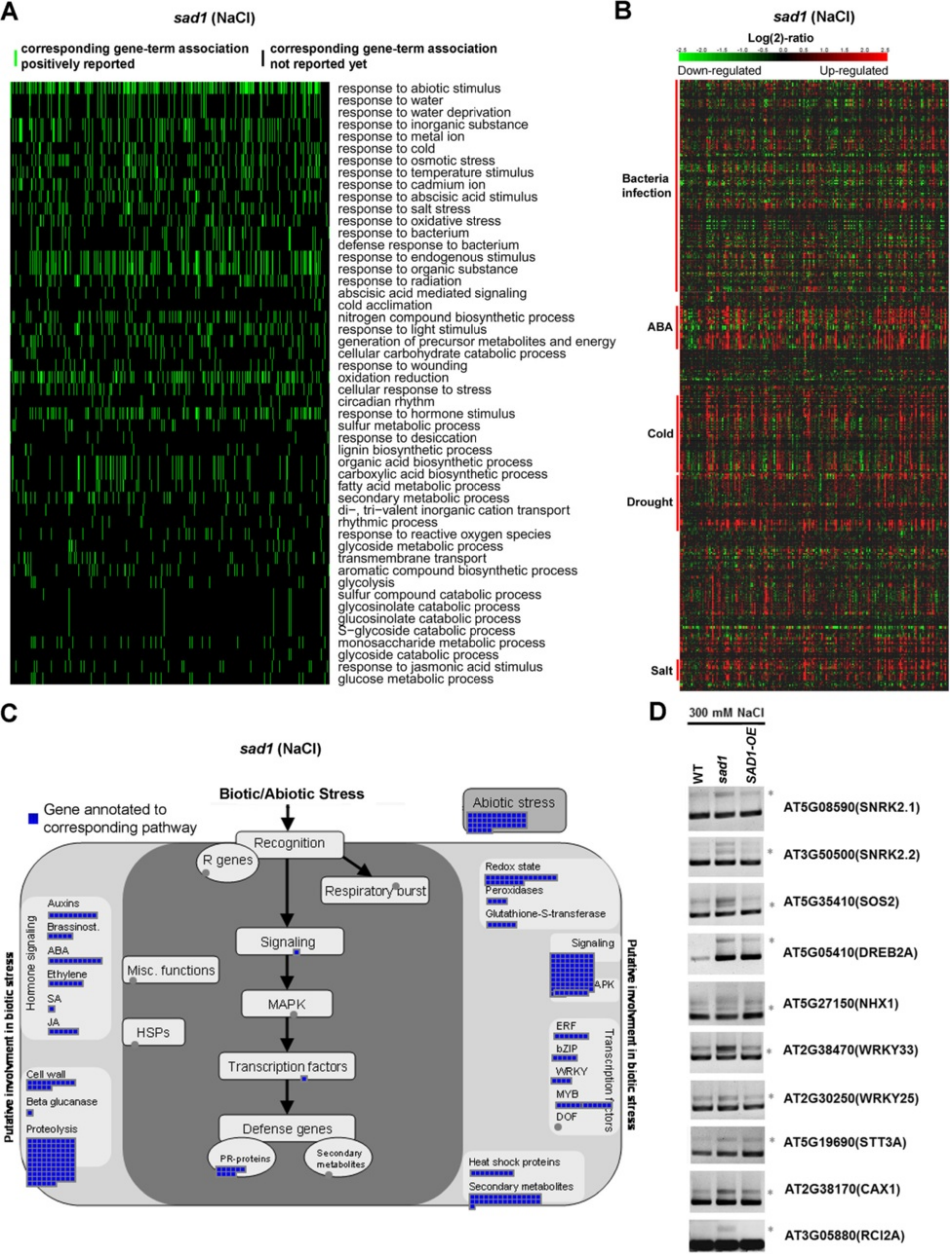
**Genes with abnormal splicing in *sad1 *are closely associated with stress response and transcriptional activation. (A)** A two-dimensional view of the relationship between the genes with abnormal splicing and their functional annotations generated by the DAVID software. The top 50 functional annotations that were ordered by the enrichment scores were selected for the two-dimensional view, which indicates that genes with abnormal splicing were strikingly enriched (colored green) in the response-to-abiotic-stress category. **(B)** A heatmap was generated by mapping the genes enriched at the response-to-abiotic-stress pathways to the microarray database using Genevestigator. The heatmap indicates that genes with abnormal splicing in *sad1* are mostly up-regulated (colored red) by ABA, cold, drought and salt stress but less regulated by biotic stress (bacteria infection). **(C)** A network generated by Mapman indicates that genes with aberrant splicing in *sad1* are involved in various stress response pathways, including hormone-signaling pathways, MAPK-signaling pathways and transcription regulation. **(D)** Validation of the intron retention in 10 stress-responsive genes by RT-PCR using the intron-flanking primers. The grey asterisks (*) denote the intron-retained splicing variants. ABA, abscisic acid; SA, salicylic acid; JA, jasmonic acid; *sad1*, *sad1* mutant; WT, wild type; HSP, heat shock protein; MAPK, mitogen-activated protein kinase; ERF, ethylene response factor; bZIP, basic-leucine zipper; WRKY, WRKY transcription factor; DOF, DNA-binding with one finger; PR-proteins, pathogenesis-related proteins; R genes, (plant disease) resistance genes.

We further analyzed functional categories and pathways of the genes with abnormal splicing in the *sad1* mutants. We identified 3,354 genes with abnormal splicing in control or NaCl-treated *sad1* mutants, the majority of which were with intron retention. Moreover, 83% of these genes were unique to either the control treatment or the NaCl treatment, suggesting that abnormal splicing may be specific to different treatments. An analysis of functional categories using the software DAVID [22,23] revealed that these abnormally spliced genes were significantly enriched at several biological processes, including response to abiotic stimulus, response to stress, photosynthesis, and protein transport, suggesting that SAD1 is involved in multiple biological processes through regulating pre-mRNA splicing (Additional files 25 and 26). Interestingly, we observed a striking enrichment at the response-to-abiotic-stress pathways, which were commonly observed in both treatments (Figure 4A; Additional file 27). Further analysis using Genevestigator [24] showed that the stress-responsive genes with abnormal splicing in NaCl-treated *sad1* mutants were closely associated with the response to salt and ABA stresses (Figure 4B); whereas, those in *sad1* under the control condition were not associated with the response to salt and ABA stresses (Additional file 28), but rather related to the response to various other environmental stresses. These results not only are consistent with the salt-sensitive phenotypes of *sad1* mutants, but also suggest that SAD1 plays critical roles in effectively regulating splicing of stress-responsive genes under stress conditions. Meanwhile, we found that genes with splicing defects coincided with those regulated by transcriptional activation under the respective treatments (shown in Figure 4B), which suggests that the occurrence of the splicing defects could follow or co-occur with transcriptional activation.

Further analysis using Mapman [25] suggested that genes with aberrant splicing in *sad1* mutants are involved in various stress response pathways, including hormone-signaling pathways, MAPK-signaling pathways and transcription regulation (Figure 4C; Additional file 29). Notably, some important genes (such as *SnRK2.1* and *2.2*, *SOS2*, *DREB2A*, *NHX1*, *WRKY33*, *WRKY25*, *STT3A*, *CAX1* and *RCI2A*) involved in stress responses were identified to have splicing defects in the *sad1* mutant. Among these genes, *SnRK2.1* and *2.2* encode members of SNF1-related protein kinases activated by ionic (salt) and non-ionic (mannitol) osmotic stress that are required for osmotic stress tolerance [26]; *SOS2* encodes a protein kinase essential for salt tolerance [27]; *DREB2A* encodes a transcription factor that activates drought and salt stress-responsive genes [28]; *NHX1* encodes a vacuolar sodium/proton antiporter whose overexpression increases salt tolerance in several plant species including *Arabidopsis*[29]; *WRKY33* and *WRKY25* encode plant WRKY transcription factors involved in response to salt and other stresses [30,31]; *STT3A* encodes an oligosaccharyl transferase whose knockout mutants are hypersensitive to high salt conditions [32]; *CAX1* encodes a high affinity vacuolar calcium antiporter and can be activated by SOS2 to integrate Ca^2+^ transport and salt tolerance [33]; and *RCI2A* (Rare-cold inducible 2A), whose product plays a role in preventing over-accumulation of excess Na^+^ and contributes to salt tolerance [34]. These genes showed increased intron retention in the mutants, which were also validated by RT-PCR using intron-flanking primers where the corresponding intron-retained transcripts were more obviously identified in *sad1*, consistent with the RNA-seq data (Figure 4D). Above all, these results suggest that genes with aberrant splicing in *sad1* are closely related to stress response, which could directly or indirectly contribute to the stress sensitivity of the *sad1* mutant.

### Overexpression of SAD1 rescues the splicing defects in the *sad1* mutant and strengthens splicing accuracy under salt stress

**Figure 5:**
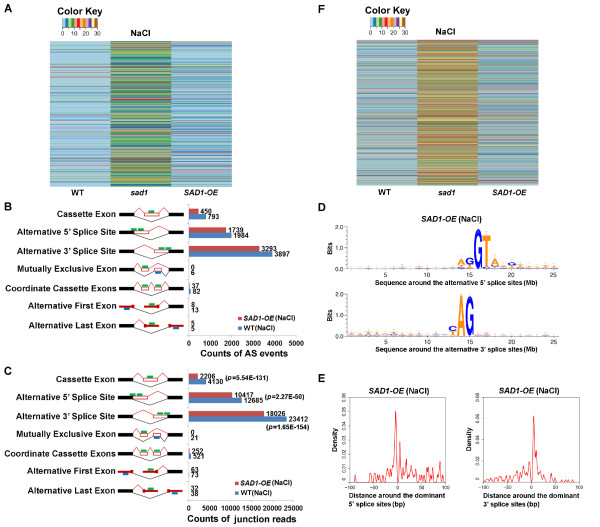
**Comparison of alternative splicing between the wild-type and SAD1-OE plants. (A)** Profiling the normalized (by total uniquely mapped reads) read coverage of the splice junctions that were over-represented in the *sad1* mutant relative to the wild type and SAD1-OE. The profiles indicate that the AS patterns in *sad1* were completely or largely restored by overexpressing *SAD1*. **(B)** The counts of each type of AS event in the wild type and SAD1-OE. The green/blue bars represent forward and reverse sequencing reads. **(C)** The total counts of the splice junction reads from each type of AS in the wild type and SAD1-OE. The *P* values were calculated by Fisher’s exact test comparing the junction read counts and the uniquely mapped reads between the wild type and SAD1-OE. **(D)** The sequences around the alternative 5′SSs and 3′SSs that were absent in the SAD1-OE were shown by Weblogo. **(E)** Distribution of activated alternative 5′SSs and 3′SSs around the dominant ones are shown. These alternative 5′SSs and 3′SSs were enriched in the downstream or upstream 10 bp region of the dominant 5′SSs and 3′SSs (position 0 on the *x*-axis), respectively. **(F)** Profiling the normalized (by total uniquely mapped reads) read coverage of the introns that were over-represented in the *sad1* mutant relative to the wild type and SAD1-OE. The profiles indicate that the intron retention in *sad1* was largely restored by overexpressing *SAD1*. AS, alternative splice; *sad1*, *sad1* mutant; SAD1-OE, plants over-expressing wild-type *SAD1* in the *sad1* mutant background; WT, wild type.

To address the question whether the splicing defects seen in *sad1* mutants result from loss of the wild-type SAD1 protein, we overexpressed the wild-type *SAD1* cDNA in the *sad1* mutant, and performed RNA-seq on the rescued plants (SAD1-OE). We first compared the expression levels of splice junctions in SAD1-OE, C24 and *sad1.* We found that the AS events previously seen in *sad1* were completely or at least partially suppressed in SAD1-OE plants (Figure 5A; Additional file 30), demonstrating that overexpression of *SAD1* was sufficient to rescue the *sad1*-dependent AS defects. While our previous study indicated that the *sad1* mutation was recessive with regard to the morphological, physiological and stress-inducible gene expression phenotypes [14], we could not rule out the possibility that an isoform of the *sad1* mutant protein (for example, isoform 3, Figure 1D) might have a dominant-negative effect that could be partly responsible for the SAD1-OE’s incomplete rescue of some of the splicing defects in *sad1*. Interestingly, when comparing the number of AS events between C24 and SAD1-OE, we found that the numbers of alternative 5′SSs, alternative 3′SSs and exon-skipping in the NaCl-treated SAD1-OE were obviously smaller than those in the corresponding C24 (Figure 5B), and the numbers of corresponding junction reads were also significantly lower (*P* <0.001) (Figure 5C). These results were not observed in the control treatment (Additional file 31). These observations indicate that overexpression of *SAD1* could inhibit AS under salt-stress conditions. Using Fisher’s exact test, we identified 454 alternative 5′SSs, alternative 3′SSs and exon-skipping events from 434 genes that were significantly absent in NaCl-treated SAD1-OE (Additional file 32). Further analyses showed that these alternative 5′SSs and 3′SSs are still associated with GU or AG dinucleotides (Figure 5D) and enriched downstream or upstream of the dominant 5′SSs and 3′SSs (Figure 5E), suggesting that overexpression of *SAD1* inhibits the usage of alternative 5′SSs and 3′SSs and promotes the usage of the dominant ones. Together with the result that SAD1-depletion activates the alternative 5′SSs and 3′SSs, we suggest that SAD1 could dynamically regulate the selection of 5′SSs and 3′SSs and control the splicing accuracy and efficiency.

We further compared the expression levels of introns in SAD1-OE with those in C24 and *sad1*. We found that the expression of most introns in SAD1-OE was restored to normal levels (Figure 5F; Additional file 33), demonstrating that the intron retention indeed resulted from the *sad1* mutation. Furthermore, using Fisher’s exact test we identified 76 introns from 75 genes that were significantly absent in NaCl-treated SAD1-OE, but were over-represented in NaCl-treated C24 (Additional file 34). This result shows that *SAD1*-overexpression can increase splicing efficiency.

### Overexpression of *SAD1* improves plant salt tolerance

**Figure 6:**
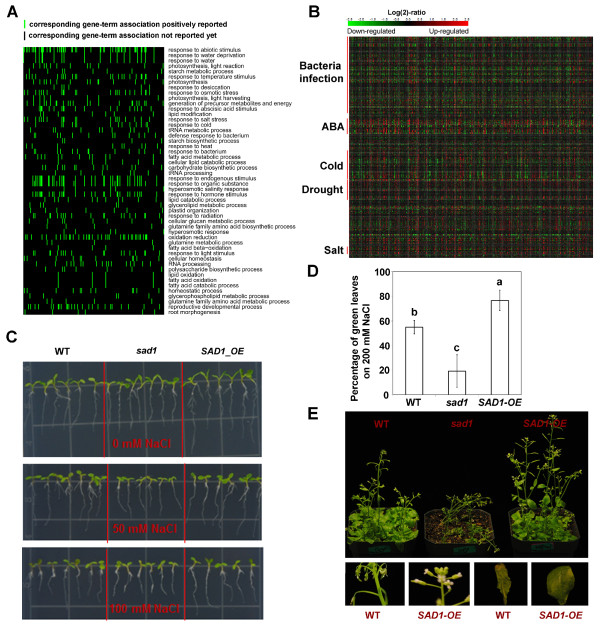
**Genes with increased splicing efficiency in SAD1-OE plants are related to stress response and overexpression of *SAD1 *improves salt stress tolerance. (A)** A two-dimensional view of the functional annotation of genes with increased splicing efficiency in SAD1-OE. The top 50 functional annotations that were ordered by the enrichment scores were selected for two-dimensional view, which indicates that genes with increased splicing efficiency were strikingly enriched (green) in the response-to-abiotic-stress pathways. **(B)** A heatmap was generated by mapping the genes enriched at the response-to-abiotic-stress pathways to the microarray database using Genevestigator. The heatmap indicates that genes with abnormal splicing are closely associated with stress responses and are up-regulated (red) by the indicated stresses. **(C)** Increased salt tolerance in seedlings overexpressing *SAD1*. Twelve-day-old seedlings on the regular ½ Murashige and Skoog (MS) medium were transferred to ½ MS media supplemented with the indicated concentrations of NaCl. The pictures were taken four days after the transfer. **(D)** Percentage of green leaves of seedlings on 200 mM NaCl media. Two-week-old seedlings grown on ½ MS media were transferred to ½ MS medium plates supplemented with 200 mM NaCl and incubated for five days before counting the number of green leaves or yellow and bleached leaves. A total 36 seedlings for each genotype were counted. Data are averages and standard deviations. Averages with different letters are statistically different (*P* <0.01, *t*-test). **(E)** Morphology of 28-day-old wild-type, *sad1* and transgenic plants (SAD1-OE) that were subjected to four-day treatment with 400 mM NaCl solution. Also shown at the bottom are pictures of the damaged inflorescent stem and leaf seen in the wild type compared to undamaged ones in the SAD1-OE. ABA, abscisic acid; *sad1*, *sad1* mutant; SAD1-OE, plants over-expressing wild-type *SAD1* in the *sad1* mutant background; WT, wild type.

In the NaCl-treated SAD1-OE plants*,* we identified 506 genes with decreased alternative 5′SSs, alternative 3′SSs, exon-skipping or intron retention. Analyses of the expression level for these genes demonstrated that their functional transcripts tended to be up-regulated in SAD1-OE plants, indicating that overexpression of *SAD1* leads to the increase of functional mRNAs (Additional file 35). Analyses of the functional categories of these genes revealed that they were strikingly enriched in the group of ′response-to-abiotic-stimulus′ (Figure 6A; Additional file 36). More specifically, these genes were well associated with the response to salt and ABA stresses and transcriptional activation (Figure 6B). Therefore, overexpression of *SAD1* can increase splicing accuracy and efficiency of stress-responsive genes under stress conditions. This result further elucidates the specific regulation of SAD1 in splicing of the stress-related genes and the potential relationship between transcription and splicing.

Further analysis suggested that these genes are involved in various stress response pathways (Additional file 37). Some of the stress-responsive genes that were more effectively spliced in SAD1-OE included *ABF3/ABF2*, encoding ABRE binding factors that mediate ABA-dependent stress responses [35,36]; *CIPK3*, encoding CBL-interacting serine/threonine-protein kinase 3 that is involved in the resistance to abiotic stresses (for example, high salt, hyperosmotic stress) by regulating the expression of several stress-inducible genes [37]; and *DREB2A*, that encodes a transcriptional factor mediating high salinity- and dehydration-inducible transcription [28]. These genes have been reported to be key regulators of ABA or salt-stress responses.

With the increased splicing efficiency in these key regulators of ABA or salt-stress responses, we were curious to know whether the SAD1-OE plants would have improved tolerance to salt stress. To test this, one-week-old seedlings of C24, *sad1* and SAD1-OE grown on the regular Murashige and Skoog (MS) agar medium were transferred to MS agar plates supplemented with 0 (control), 50, 100 or 200 mM NaCl. We found that SAD1-OE seedlings showed enhanced tolerance to 100 mM NaCl on vertically placed plates (Figure 6C). At 200 mM NaCl, however, root elongation of all genotypes was inhibited and seedlings were not able to survive an extended period of the stress treatment (data not shown). Measuring the root growth of the seedlings showed that the roots of SAD1-OE were longer than those of C24 and *sad1* at 100 mM NaCl (Additional file 38). We also tested salt tolerance of seedlings on horizontally placed agar medium plates. Two-week-old seedlings from ½ MS media were transferred onto 200 mM NaCl media and incubated for five days. The percentage of green leaf number over total leaf number was calculated for each seedling. The data indicated that SAD1-OE seedlings had a higher percentage of green leaves, suggesting that they were significantly less damaged by the salt stress than were the wild-type or *sad1* seedlings (Figure 6D). To test further whether SAD1-OE plants were tolerant to salt stress at the adult stage and in soil, we grew these seedlings in soil and irrigated with either 50, 100, 150, 200 or 400 mM NaCl solutions at intervals of four days (see Methods). After two weeks of treatment, we found that *sad1* plants were very sensitive to salt stress at concentrations above 150 mM and wild-type seedlings also exhibited signs of damages at higher salt concentrations as indicated by wilty inflorescence and damaged leaves, whereas the SAD1-OE plants were not obviously affected by the stress treatment and were also taller than the wild-type plants (Figure 6E; Additional file 39). These results indicate that SAD1-overexpression improves salt tolerance, which correlates with increased splicing accuracy and efficiency of stress-responsive genes.

## Discussion

Although studies in other eukaryotes, and more recently in plants, have demonstrated that LSm proteins 2-8, as the core of U6 RNPs, function in pre-mRNA splicing, whether or not these proteins have any roles in regulation of splicing efficiency and selection of splice sites has not yet been determined. In this study, through comprehensive transcriptome analysis of mutant and transgenic plants overexpressing the LSm5 gene *SAD1*, we demonstrated that SAD1 could dynamically regulate splicing efficiency and selection of splice sites in *Arabidopsis*. We also revealed that SAD1 modulates splicing of stress-responsive genes under salt-stress conditions. Finally, we showed that overexpression of *SAD1* significantly improved splicing efficiency of the salt-responsive genes and resulted in enhanced salt tolerance in transgenic plants.

We found that SAD1 depletion activated the alternative 5′SSs and 3′SSs proximal to the dominant ones, suggesting that the wild-type SAD1 protein is necessary for precise splice-site recognition. To our surprise, relative to the wild-type plants, overexpression of SAD1 can strengthen the recognition accuracy and globally inhibit AS under salt-stress conditions. Therefore, we conclude that SAD1 can control selection of splice sites and splicing efficiency in a manner depending on SAD1’s abundance. This kind of splicing regulation, which could be referred to as a dynamic model, differs from but complements the kinetic model of splicing regulation [1,38,39]. In the kinetic model, the elongation rate of RNA polymerase II (Pol II) affects splicing efficiency such that a slower Pol II would allow more time for the recognition and processing of weak splicing sites so that splicing efficiency is enhanced. In the dynamic model, we reasoned that the spliceosome or other complexes involved in splicing are under thermodynamic equilibrium between association (complex formation) and disassociation (complex breakdown) at any given condition. A higher dosage of certain key small nuclear RNPs or splicing factors may drive the reaction toward the formation of the complex to enhance splicing efficiency. This dosage-dependent control of splicing suggests an alternative splicing regulation and it may be particularly important for the splicing of particular group of genes such as stress-inducible genes as discussed below.

Whereas increased alternative 5′SS usage was seen in *sad1* both under control and salt-stress conditions (Figures 2A,B; Additional file 9A,B), the increase of alternative 3′SSs caused by SAD1 depletion and the inhibition of AS caused by *SAD1* overexpression were only observed under salt-stress conditions. These findings indicate that SAD1 depletion or overexpression appears to impact splicing under salt-stress conditions more than under normal conditions. We considered that this distinct impact of SAD1 on splicing under normal versus stress conditions might have to do with increased transcriptional activation of the stress-responsive genes. Under salt-stress or other abiotic-stress conditions, plants activate the expression of a large number of stress-responsive genes that are not expressed or are expressed at lower levels under normal non-stressful conditions [40,41]. With the simultaneous production of a large amount of these stress-inducible pre-mRNAs, cells would need to immediately recruit a significant amount of splicing factors and other factors for their co-transcriptional or post-transcriptional processing. This imposes a huge burden on the splicing machinery and as a result a significant portion of these transcripts fail to be processed adequately when the splicing machinery is compromised. This may be the reason why most of the splicing defective genes in *sad1* are stress-regulated (Figure 4). Conversely, a higher SAD1 dosage could play a dominant role in enhancing the splicing efficiency of these salt-responsive genes through the promotion of recruitment and assembly of the splicing machinery as discussed above. As a result, the change in the AS pattern in SAD1-OE plants was more obvious under salt-stress conditions than under control conditions. Thus, the expression of these (and other) highly inducible genes may be particularly subjected to the dynamic regulation by certain splicing factors, which to some extent is similar to the kinetic regulation of splicing, both reflecting the saturated capability of cellular machinery.

We thought that the decreased splicing efficiency of the stress-responsive genes might contribute to the stress-sensitivity of the *sad1* mutant. The splicing defects in *sad1* lead to widespread intron retention in many stress-responsive genes (317 genes, Additional file 40). These genes include those encoding known key determinants of salt tolerance such as *SnRK2.1/2.2*, *SOS2*, *DREB2A*, *NHX1*, *WRKY33*, *WRKY25*, *STT3A*, *CAX1* and *RCI2A*. The expression level of the functional transcripts for many of these genes were also found to be down-regulated (Additional file 41), although the cause of this down-regulation is unclear. All of these intron-containing transcripts were predicted to generate premature stop codons and truncated proteins if translated. This large-scale ‘hidden’ change in pre-mRNA splicing efficiency or gene expression, although relatively small for some of the individual genes, may collectively undermine plant’s readiness for the stress. However, it should be pointed that a direct relationship between the splicing defects and stress sensitivity in the *sad1* mutant could not be established at this point.

Interestingly, an increase of splicing efficiency and expression of stress-responsive genes correlated with improved stress tolerance of the plants. Indeed, transgenic plants overexpressing *SAD1* exhibited obviously increased tolerance to salt stress (Figure 6E), although the magnitude of the increase was moderate. Nonetheless, this finding is very significant for two reasons. First, it indicates that splicing efficiency may play an important role in regulating plant stress resistance. This is consistent with findings in several other genetic studies, where certain RNA processing factors were also found to be required for plant stress resistance. These factors include, for example, *ABH1*[42], *LOS4*[43] and *RCF1*[44], although the mechanisms involved were unclear. Secondly, our finding provides a new approach to improving plant stress resistance, namely, by regulating the splicing efficiency. Current methods to increase plant salt tolerance mostly involve the overexpression of structure genes such as ion transporter genes [29,45,46]. Constitutively expressing these structure genes may cause unwanted side effects that would result in reduced yield under normal growth conditions. However, enhancing splicing efficiency does not affect gene expression under normal conditions, as demonstrated in this study. Our finding may also be applicable to enhancing stress tolerance or other traits in other eukaryotic systems.

## Conclusions

We demonstrated that SAD1 dynamically regulates splicing efficiency and plays a regulatory role in the selection of splice sites. Furthermore, we found that SAD1 specifically modulates splicing of the stress-responsive genes under stress conditions. Finally, we showed that overexpression of *SAD1* improves salt tolerance of transgenic plant, which correlates with the increased splicing efficiency of the salt-stress-responsive genes. Our study provided novel insights into the regulatory role of SAD1 or LSm proteins in splicing and also suggested new strategies to improving splicing efficiency and bioengineering stress-resistant plants.

## Materials and methods

### Plant materials and growth conditions

The *Arabidopsis sad1* mutant in the C24 background was described previously [14]. For overexpressing the *SAD1* gene, the *SAD1* cDNA, amplified from the wild-type plant, was cloned into pENTRY1A. The LR reaction was then performed between pGWB502 and the pENTRY1A-SAD1. The resulting plasmid (pGWB502-SAD1) was introduced into *Agrobacterium tumefaciens* GV3101 and transformed into *sad1* mutant plants using the floral dipping method. The transformants were selected on a ½ MS medium supplemented with 25 μg/ml hygromycin. Positive transformants were further confirmed by genotyping using the primers 5-CACCGGATCCTGATGGCGAACAATCCTTCACAGC-3; 5-TAATGAATTCGATCATTCTCCATCTTCGGGAGACC-3 for *SAD1* cDNA. The confirmed transgenic seedlings (referred to as SAD1-OE) were used for RNA sequencing and RT-PCR analyses.

Seeds of C24, *sad1* and SAD1-OE plants were sterilized with 50% bleach and 0.01% Triton X-100. The sterilized seeds were sown on ½ MS plates supplemented with 3% sucrose. After four-day stratification at 4°C, the plates were placed under a 16 h-light and 8 h-dark cycle at 21°C for germination and seedling growth. Twelve days later, the seedlings were treated with H_2_O (control) or 300 mM NaCl for 3 h, and harvested for total RNA extraction.

### RNA extraction, library construction and sequencing

Using the TRIzol Reagent (15596–026, Invitrogen Co., Carlsbad, CA, USA), total RNAs were extracted from 12-day-old seedlings of wild-type C24, *sad1* and SAD1-OE. Polyadenylated RNAs were isolated using the Oligotex mRNA Midi Kit (70042, Qiagen Inc., Valencia, CA, USA). The RNA-seq libraries were constructed using Illumina Whole Transcriptome Analysis Kit following the standard protocol (Illumina, HiSeq system) and sequenced on the HiSeq platform to generate high-quality single-end reads of 101 nucleotides (some with 85 nucleotides due to machine failure) in length.

### RNA-sequencing data analysis pipeline

To analyze RNA-seq data, a pipeline was developed, which involved five steps: read alignment and junction prediction, the filter of false positive junctions, annotation of AS events, global comparison of AS and the identification of differential AS events (for details, see Additional file 42).

#### Read alignment and junction prediction

TopHat [21] was used to align the reads against the *Arabidopsis* genome sequences and annotated gene models were downloaded from TAIR10 [18] allowing two nucleotide mismatches. Meanwhile, TopHat was also used to predict the splice junctions that did not permit any mismatches in the anchor region of a spliced alignment. The splice junctions were classified into known and novel splice junctions using the Perl script, which takes as input genome coordinates of all annotated exons and all predicted splice junctions. In addition, the expression levels of transcripts were measured by reads per kilo base per million values using the Cufflinks software [47].

#### The filter of false positive junctions

To estimate thresholds for filtering false positive junctions, two datasets of random and annotated splice junctions were first created. The dataset of 80,000 random splice junctions was created by joining each annotated 5′ donor sequence (90/74 bp from 5′SSs) and the annotated 3′ donor sequence (90/74 bp from 3′SSs) located on a different chromosome (Additional file 42). The 119,618 annotated splice junctions were created by joining each annotated 5′ donor sequence (90/74 bp from 5′SSs) and the annotated 3′ donor sequence (90/74 bp from 3′SSs) in order based on the gene annotation (Additional file 42). All splice junctions contained 90/74 nucleotides of exon sequence on either side of the junction to force an alignment overhang of at least 11 nucleotides from one side of the splice junction to the other. Then, the mapping software BWA [48] was used to align all reads to the random and annotated splice junctions that did not permit any mismatches. The alignments to random junctions were considered to be false positives, because such junctions are thought to rarely exist when compared to annotated junctions. We further characterized the false positive junctions, which generally have an overhang size of fewer than 20 bp and lower read coverage (Additional file 7A-B). To minimize the false positive rate, the overhang size with more than 20 bp and at least two reads spanning the junctions were required as cutoff value to filter the false positive junctions.

#### Annotation of alternative splicing events

JuncBASE [49] was used for annotating all AS events, including cassette exons, alternative 5′SSs, alternative 3′SSs, mutually exclusive exons, coordinate cassette exons, alternative first exons, alternative last exons and intron retention, which takes as input genome coordinates of all annotated exons and all confidently identified splice junctions. Notably, for identifying the events of intron retention, we required that at least five reads covered at least 50% of the region of one intron.

#### Global comparison of alternative splicing

The global comparison of AS among WT, *sad1* and SAD1-OE was started with equally and randomly re-sampling uniquely-mapped reads to make sure that the comparison was at the same level. The comparison refers to the two facets: the absolute amount of each type of AS event and the number of junction reads that were assigned to each type of AS event, because both of them can be used to measure the global changes of AS. Meanwhile, Fisher’s exact tests in R [50] were used to identify differential representation of each type of AS event, performed on the number of junction reads that were assigned to each type of AS event.

#### The identification of differential alternative splicing events

Fisher’s exact tests were also used to identify differential representation of each AS event. For alternative 5′SSs and 3′SSs and exon-skipping events, Fisher’s exact tests were performed on the comparison of the junction-read counts and the corresponding exon-read counts between C24 and *sad1* or SAD1-OE. The events with *P* <0.01 were identified as significantly different events. In addition, for those AS events that were uniquely identified in C24, *sad1* or SAD1-OE, we would consider them significant if there were at least five junction reads to support and the *P* value of these events was assigned to equal zero. Similarly, for intron retention, Fisher’s exact tests were performed on the intron-read counts and the corresponding exon-read counts between C24 and *sad1* or SAD1-OE. The events with *P* <0.001 were identified as significantly differential events. In addition, for those intron retention uniquely identified in C24 or the mutant, we would consider them significant if there was at least five-time coverage to support and the *P* value of these events was assigned to equal to zero.

### RT-PCR validation

The selected AS and intron retention events were validated by RT-PCR using a set of primers (Additional file 43) that were designed based on each AS event. Total RNAs from the C24, *sad1* and SAD1-OE plants were extracted using Trizol solution (Invitrogen; cat.10837-08), treated with DNAase I, and reverse-transcribed to cDNA (random priming) by using a standard protocol (SuperScript II reverse-transcriptase, Invitrogen).

### Quantitative RT-PCR

For the RT reaction, we used 3 μg total RNAs from the control (H_2_O) and 300 mM NaCl-treated C24, *sad1* and SAD1-OE seedlings. The RT reactions were done with the Invitrogen SuperScript® III First-Strand Synthesis SuperMix in a 20 μl reaction system; the random Hexamer was used for first strand synthesis. The RT-solution was diluted 10 times, and 1 μl of the solution was used as template in 10 μl reaction system with 2 × SYBR Green (Invitrogen) Supermix (ROX). The quantitative RT-PCRs were performed in triplicate using the ABI 7900HT Fast Real-Time PCR System (Applied Biosystems Inc., Foster City, CA, USA). The primers 5-AAGGAGATAAG-GAGCTCGTTGG-3 and 5-ATCTGATCAAGCTTTGTGACC-3 were used for detecting expression levels of *SAD1*.

### Salt-stress tolerance assays

Surface-sterilized seeds of C24, *sad1* and SAD1-OE were sown onto agar plates with ½ MS and 1.2% agar. The plates were then kept at 4°C for four days before being incubated at 21°C for germination. Four days after germination, the seedlings were transferred to ½ MS agar plates supplemented with 0, 50, 100 or 200 mM NaCl, respectively. The seedlings were then allowed to grow for four days, and seedlings were photographed. Root length of these seedlings was measured by using Image J [51]. For measuring leaf damage, two-week-old seedlings grown on ½ MS plates (0.6% agar) were transferred onto ½ MS medium plates supplemented with 200 mM NaCl and incubated for five days. The number of green leaves and yellowish or bleached leaves was counted for each seedling and percentage of green leaves among total leaves was calculated (leaves of the no-salt control treatment were all green and were not counted). To further test the tolerance to salt stress, seedlings grown on ½ MS agar plates were transferred to soil. One week after the transfer, the seedlings were irrigated with 50, 100 or 150 mM NaCl (in 1/8 MS salt), respectively [29]. At 24 days after the transfer, the plants were further irrigated with 400 mM NaCl (100 ml) and pictures were taken four days later.

### Data availability

The RNA-seq data generated in this work has been submitted to the Sequence Read Archive database in NCBI. The accession number is SRP026082.

## Abbreviations

3′SSs: 3′ splice sites; 5′SSs: 5′ splice sites; ABA: abscisic acid; AS: alternative splicing; bp: base pairs; LSm: Sm-like protein; MS: Murashige and Skoog; PCR: polymerase chain reaction; RNA-seq: RNA-sequencing; RNP: ribonucleoprotein; RT: reverse transcriptase.

## Competing interests

The authors declare that they have no competing interests.

## Authors’ contributions

LX and PC conceived the project idea. PC and FD performed data analysis. SZ and SA did the experiments. PC and LX wrote the manuscript. All authors read and approved the final manuscript.

## Supplementary Material

Additional file 1 Mapping results of RNA-seq reads. Click here for file

Additional file 2 **Distribution of the RNA-seq reads along annotated *Arabidopsis *** **genomic features. Among reads that unambiguously match the *Arabidopsis *** **genome, more than 90% of reads match to annotated exons.** Click here for file

Additional file 3 **Distribution of the RNA-seq read coverage was plotted along the length of the transcriptional unit.***x*-axis indicates the relative length of transcripts, and *y*-axis shows the median depth of coverage. Click here for file

Additional file 4 **Saturation curve for gene detection.** Randomly sampled reads were plotted against the expressed genes. *x*-axis shows the number of the mapped reads and *y*-axis displays the number of the expressed genes. Click here for file

Additional file 5 **Transcription profiles were plotted across the *Arabidopsis *genome.** Distribution of RNA-seq read density along chromosome length is shown. Each vertical blue bar represents log2 of the frequency of reads plotted against chromosome coordinates. A schematic drawing of the chromosome and its features is shown below the read density. Approximate boundaries of centromeres are depicted in gray. Click here for file

Additional file 6 The distinctive features between known and novel splice junctions. **(A)** After comparing all the splice junctions to the gene annotation, about 83% of total junctions belong to the annotated junctions, and the remaining 17% were assigned to novel junctions. **(B)** The density of overhang size with exon for known and novel splice junctions in each sample. *x*-axis indicates the size of overhang on exon and *y-*axis indicates the density of the sizes. **(C)** The density of junction read coverage for known and novel junctions. Click here for file

Additional file 7 **The features of false positive (random) and annotated junctions. (A)** The density of the overhang size of false positive and annotated junctions. Most of false positive junctions show shorter overhang sizes, while the annotated junctions have larger overhang sizes. **(B)** The density of junction read coverage of false positives and annotated junctions. More than half of false positive junctions have only one read spanning the junction, while the annotated junctions have higher reads coverage. **(C)** Distinguishing true junctions from false positive alignments. To reduce the number of false positive junctions, as determined by randomly generated junctions, we required that the overhang size must be more than 20 bp (>20 bp) and at least two reads (>1 read) span the junctions. Using both criteria, the false positive junctions sharply reduced to very low levels (close to zero). By contrast, the annotated junctions show no obvious decrease. Click here for file

Additional file 8 **Annotation of AS events based on all the confident junctions.** The AS events include cassette exons, alternative 5′SSs, alternative 3′SSs, mutually exclusive exons, coordinate cassette exons, alternative first exons and alternative last exons. Click here for file

Additional file 9 **Comparison of global AS between the wild type and *sad1 *under the control conditions. (A)** The counts of each type of AS events in the wild type and *sad1*. The green/blue bars represent forward and reverse sequencing reads. **(B)** The total counts of the splice junction reads from each type of AS in the wild type and *sad1*. The *P* values were calculated by Fisher’s exact test comparing the junction read counts and the uniquely mapped reads between the wild type and *sad1*. Click here for file

Additional file 10 **List of alternative 5′ splice sites that were over-represented in *sad1 *** **under the control or NaCl treatment.** Click here for file

Additional file 11 **List of exon-skipping events that were over-represented in *sad1 *** **under the control or NaCl treatment.** Click here for file

Additional file 12 List of alternative 5′ splice sites that were over-represented in the wild type under the control or NaCl treatment. Click here for file

Additional file 13 List of exon-skipping events that were over-represented in the wild type under the control or NaCl treatment. Click here for file

Additional file 14 **List of alternative 3′ splice sites that were over-represented in the NaCl-treated *sad1 *** **mutant.** Click here for file

Additional file 15 List of alternative 3′ splice sites that were over-represented in the NaCl-treated wild type. Click here for file

Additional file 16 **Validation of the AS events in 19 genes by RT-PCR and corresponding IGV visualization.** These 19 events include 9 alternative 5′SSs, 4 alternative 3′SSs and 6 exon-skipping events. For the validation of alternative 5′SSs and 3′SSs, there was only one band that represents the alternative-splice isoform, which was obviously detected in *sad1* mutants, but not or only weakly detected in the wild type and SAD1-OE. For exon-skipping events, the alternatively spliced forms were marked with grey asterisks (*). For IGV visualization, alternative splicing sites were marked by red arrows and highlighted by red arcs. Click here for file

Additional file 17 **The characters of activated alternative 5′SSs in *sad1 *under the control conditions. (A)** The sequences around the alternative 5′SSs that were over-represented in the mutant were shown by Weblogo. **(B)** Distribution of activated alternative 5′SS around the dominant ones. These alternative 5′SSs were enriched in the downstream or upstream 10 bp region of the dominant 5′SSs (position 0 on the *x*- axis). Click here for file

Additional file 18 **Comparison of intron retention between the wild-type and *sad1 *plants under the control conditions.** The RPKM values for the exons and introns were plotted. The expression of introns, but not exons, in the *sad1* mutant showed a global up-regulation. Click here for file

Additional file 19 **Validation of the intron retention in selected genes by RT-PCR using the intron-flanking primers.** The intron retained splicing variants were marked by grey asterisks (*). Click here for file

Additional file 20 **List of genes with intron retention in *sad1 *** **under the control or NaCl treatment.** Click here for file

Additional file 21 List of genes with intron retention in the wild type under the control or NaCl treatment. Click here for file

Additional file 22 **Distribution of the proportions of intron-retained transcripts to the total transcripts for each gene with intron-retention in the *sad1 *mutants.** The percentage is calculated by dividing the RPKM value of the retained intron by the RPKM value of the two-flanking exons. Click here for file

Additional file 23 **Comparison of the total transcripts and functional transcripts (without introns) between the wild type and *sad1*.** The relative expression of total transcripts was measured as the read number of the two exons flanking the retained intron, and the relative expression of functional transcripts was calculated by deducting the expression of the retained intron (measured by the read number of the retained intron) from the expression of the total transcripts. The expression levels of the total transcripts did not show obvious change between the wild types and *sad1*, but the functional transcripts tended to be down-regulated in the control and NaCl-treated *sad1* mutants. Click here for file

Additional file 24 Expression level of functional transcripts in intron-retained genes. Click here for file

Additional file 25 **Functional category of genes with abnormal splicing in the NaCl-treated *sad1 *** **mutant.** Click here for file

Additional file 26 **Functional category of genes with abnormal splicing in the *sad1 *** **mutant under the control conditions.** Click here for file

Additional file 27 **A two-dimension view of the functional annotations of genes with abnormal splicing in *sad1 *under the control conditions.** The functional classification of genes was done by using the DAVID software. The top 50 functional annotations ordered by the enrichment scores were selected for the two-dimensional view, which indicates that genes with abnormal splicing were strikingly enriched in the response-to-abiotic-stress category. Click here for file

Additional file 28 **A heatmap was generated by mapping the genes enriched at the response-to-abiotic-stress pathways to the microarray database at Genevestigator.** The heatmap indicates that genes with abnormal splicing in the *sad1* control treatment are not specifically related to the response to salt and ABA stress, but rather associated with random responses to various environmental stresses. Click here for file

Additional file 29 **A network generated by Mapman indicates that genes with aberrant splicing in the *sad1 *** **control treatment are involved in various stress response pathways, including hormone-signaling pathways, MAPK-signaling pathways and transcription regulation.** Click here for file

Additional file 30 **Profiling the normalized (by total uniquely mapped reads) read coverage of the splice junctions that were over-represented in the *sad1 *mutant relative to the wild type and SAD1-OE under the control conditions.** The profiles indicate that the AS patterns in *sad1* were completely or largely restored by overexpressing *SAD1*. Click here for file

Additional file 31 **Number of each type of AS event in the wild type and SAD1-OE under the control conditions.** Note that the numbers of alternative 5′SSs, alternative 3′SSs and exon-skipping events in SAD1-OE are close to those in the wild type. The green/blue bars represent forward and reverse sequencing reads. Click here for file

Additional file 32 List of AS events that were significantly absent in the NaCl-treated SAD1-OE plants. Click here for file

Additional file 33 **Profiling the normalized (by total uniquely mapped reads) read coverage of the introns that were over-represented in the *sad1 *mutant relative to the wild type and SAD1-OE under the control conditions.** The profiles indicate that the intron retention events in *sad1* were completely or largely restored by overexpressing *SAD1*. Click here for file

Additional file 34 List of introns that were significantly absent in the NaCl-treated SAD1-OE compared to the wild type. Click here for file

Additional file 35 **Comparison of the total transcripts and functional transcripts (without retained intron) between the NaCl-treated wild-type and SAD1-OE plants.** The reads number (log10) for the total transcripts and functional transcripts were plotted between the wild type and SAD1-OE. The functional transcripts tended to be up-regulated in the NaCl-treated SAD1-OE plants. Click here for file

Additional file 36 Functional category of genes with increased splicing efficiency in the NaCl-treated SAD1-OE plants. Click here for file

Additional file 37 A network generated by Mapman indicates that genes with increased splicing efficiency in SAD1-OE are involved in various stress response pathways, including hormone-signaling pathways, MAPK-signaling pathways and transcription regulation. Click here for file

Additional file 38 **Relative root length of the wild-type, *sad1 *and SAD1-OE seedlings after four days growth on 0, 50 or 100 mM NaCl.** Data are means and standard errors from about 15 seedlings. One-week-old seedlings grown on ½ MS medium plates were transferred to ½ MS medium plates supplemented with the indicated concentrations of NaCl and allowed to grow for four days before measuring the root length. Click here for file

Additional file 39 **Morphology of 28-day-old wild-type, *sad1 *** **and transgenic (SAD1-OE) plants grown under normal conditions (without NaCl treatments).** Click here for file

Additional file 40 **Functional category of stress-responsive genes with intron retention in the *sad1 *** **mutant.** Click here for file

Additional file 41 List of intron-retained genes that are stress-related and are down-regulated in the expression level of the functional transcripts. Click here for file

Additional file 42 **The pipeline of RNA-seq data analysis in this study.** The pipeline involves five steps: read alignment and junction prediction, the filter of false positive junctions, annotation of AS events, global comparison of AS and the identification of differential AS events. To estimate thresholds for filtering false positive junctions, two datasets of random and annotated splice junctions were created. The random splice junctions dataset was created by joining each annotated 5′ donor sequence (90/74 bp from 5′SSs) and the annotated 3′ donor sequence (90/74 bp from 3′SSs) located on a different chromosome. The annotated splice junctions dataset was created by joining each annotated 5′ donor sequence (90/74 bp from 5′SSs) and the annotated 3′ donor sequence (90/74 bp from 3′SSs) in order based on the gene annotation. Click here for file

Additional file 43 The primers used for RT-PCR to validate 22 AS events and 20 intron retention events. Click here for file
