# Supplementary figures and images for: Dynamic regulation of genome-wide pre-mRNA splicing and stress tolerance by the Sm-like protein LSm5 in Arabidopsis
Source: Genome Biol. 2014 Jan 7;15(1):R1. doi: 10.1186/gb-2014-15-1-r1 (PMC4053965; doi:10.1186/gb-2014-15-1-r1)

**WT (NaCl)**

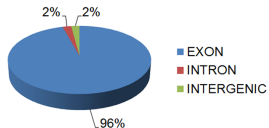

***sad1* (NaCl)**

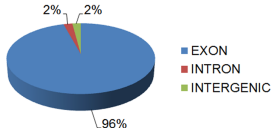

***SAD1*-OE (NaCl)**

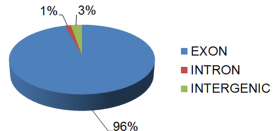

**WT (Control)**

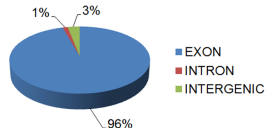

***sad1* (Control)**

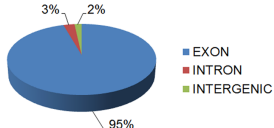

***SAD1*-OE (Control)**

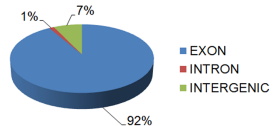

Supplement: Additional file 2 — Distribution of the RNA-seq reads along annotated Arabidopsis genomic features. Among reads that unambiguously match the Arabidopsis genome, more than 90% of reads match to annotated exons. [file gb-2014-15-1-r1-S2.pdf]

## NaCl

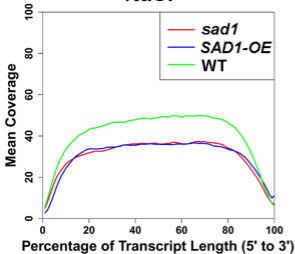

## Control

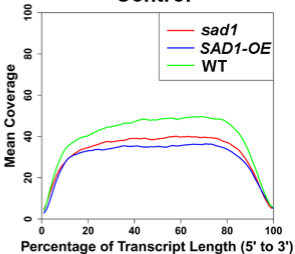

Supplement: Additional file 3 — Distribution of the RNA-seq read coverage was plotted along the length of the transcriptional unit.x-axis indicates the relative length of transcripts, and y-axis shows the median depth of coverage. [file gb-2014-15-1-r1-S3.pdf]

## NaCl

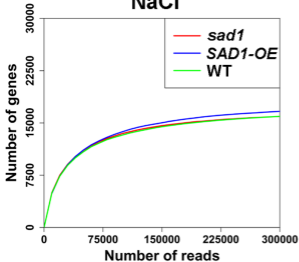

## Control

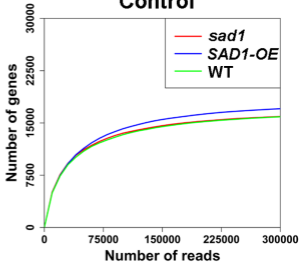

Supplement: Additional file 4 — Saturation curve for gene detection. Randomly sampled reads were plotted against the expressed genes. x-axis shows the number of the mapped reads and y-axis displays the number of the expressed genes. [file gb-2014-15-1-r1-S4.pdf]

**WT (NaCl)**

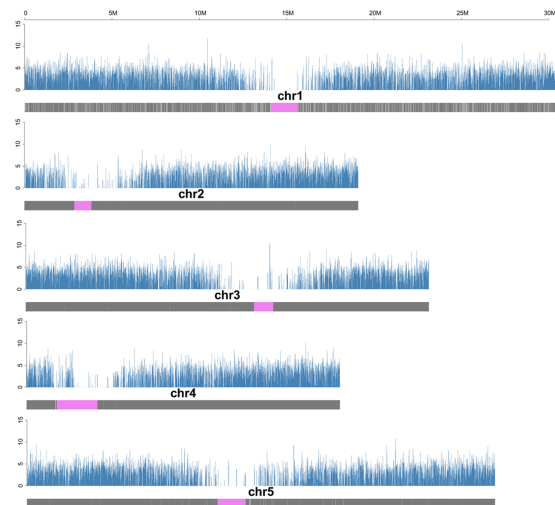

**WT (Control)**

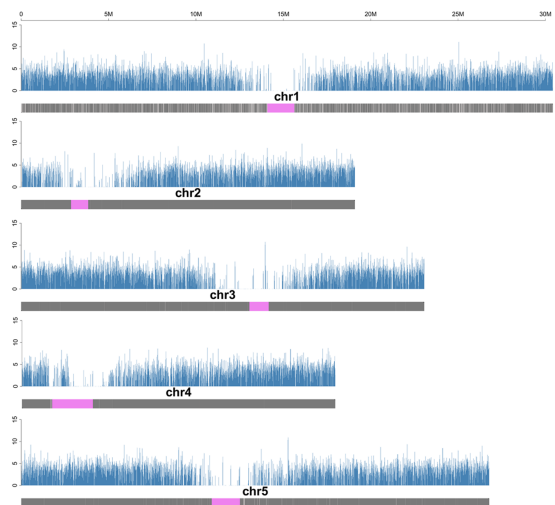

**sad1 (NaCl)**

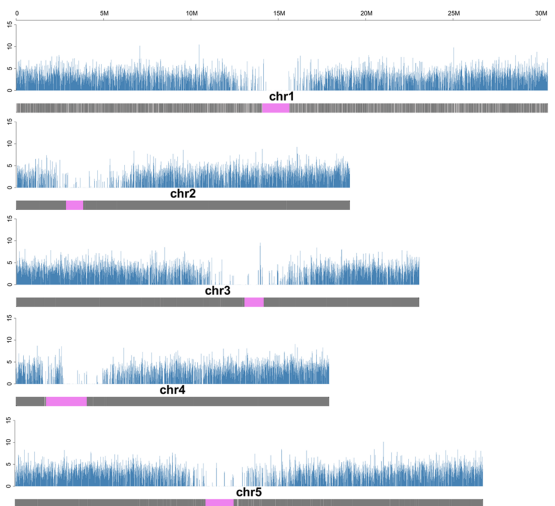

**sad1 (Control)**

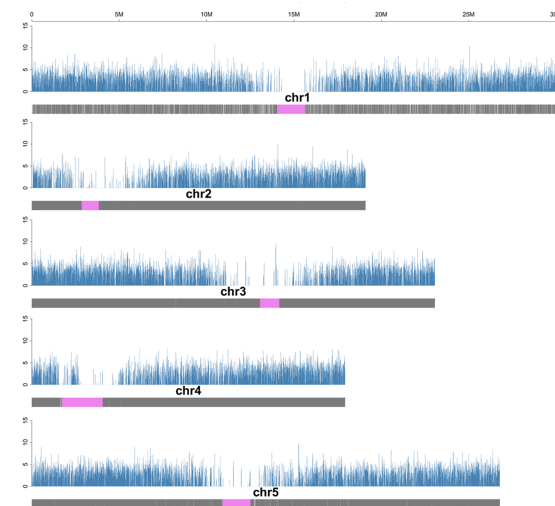

**SAD1-OE (NaCl)**

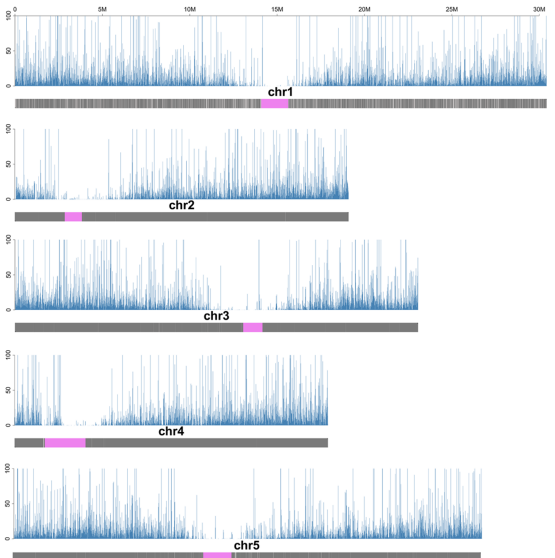

**SAD1-OE (Control)**

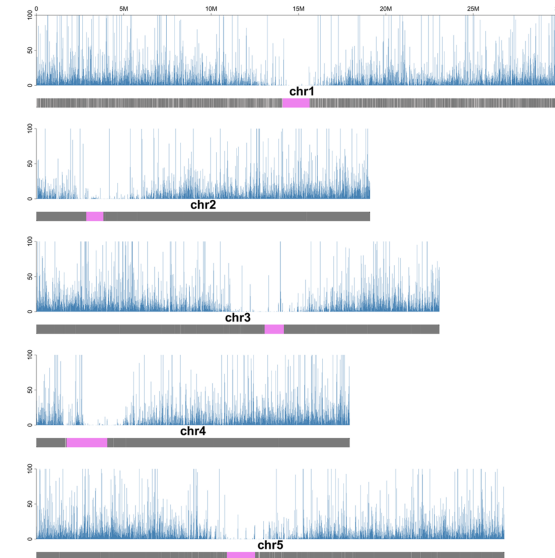

Supplement: Additional file 5 — Transcription profiles were plotted across the Arabidopsis genome. Distribution of RNA-seq read density along chromosome length is shown. Each vertical blue bar represents log2 of the frequency of reads plotted against chromosome coordinates. A schematic drawing of the chromosome and its features is shown below the read density. Approximate boundaries of centromeres are depicted in gray. [file gb-2014-15-1-r1-S5.pdf]

**A**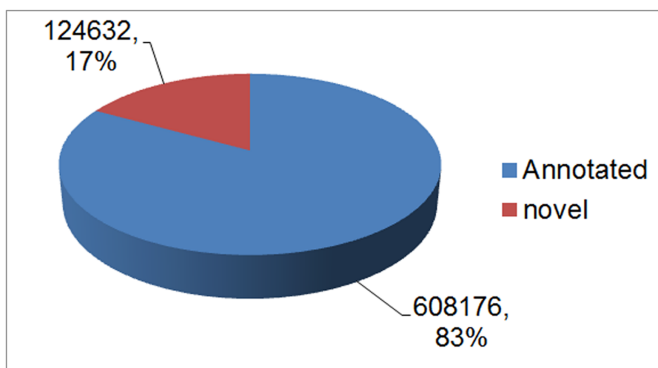**B**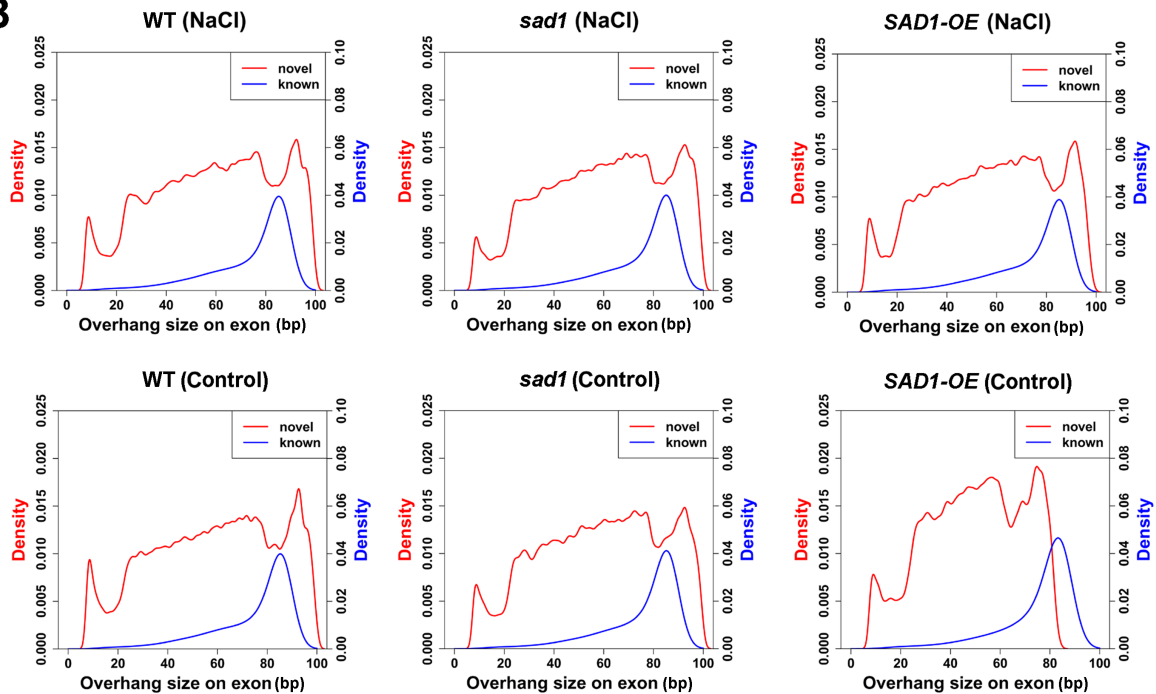**C**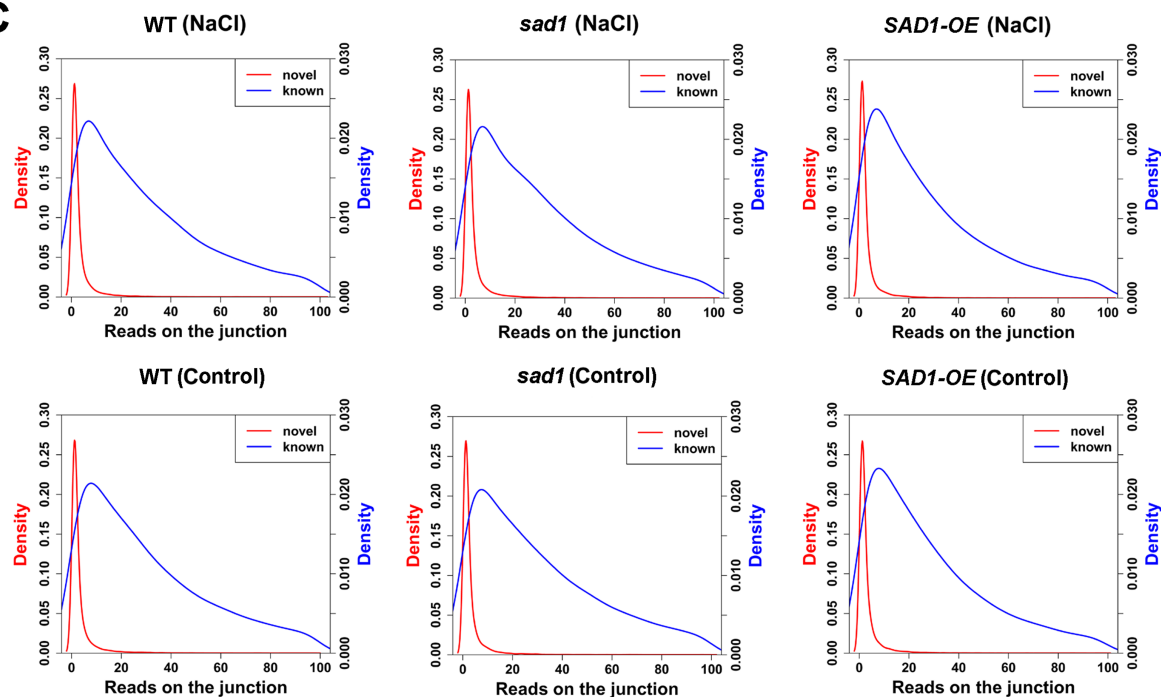

Supplement: Additional file 6 — The distinctive features between known and novel splice junctions. (A) After comparing all the splice junctions to the gene annotation, about 83% of total junctions belong to the annotated junctions, and the remaining 17% were assigned to novel junctions. (B) The density of overhang size with exon for known and novel splice junctions in each sample. x-axis indicates the size of overhang on exon and y-axis indicates the density of the sizes. (C) The density of junction read coverage for known and novel junctions. [file gb-2014-15-1-r1-S6.pdf]

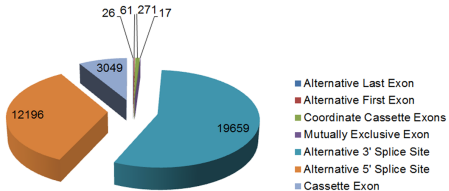

Supplement: Additional file 8 — Annotation of AS events based on all the confident junctions. The AS events include cassette exons, alternative 5′SSs, alternative 3′SSs, mutually exclusive exons, coordinate cassette exons, alternative first exons and alternative last exons. [file gb-2014-15-1-r1-S8.pdf]

**A**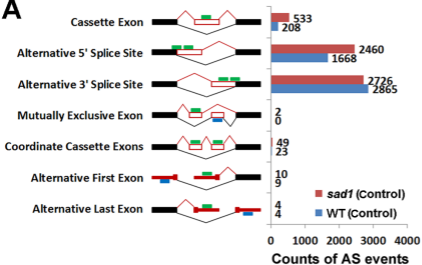**B**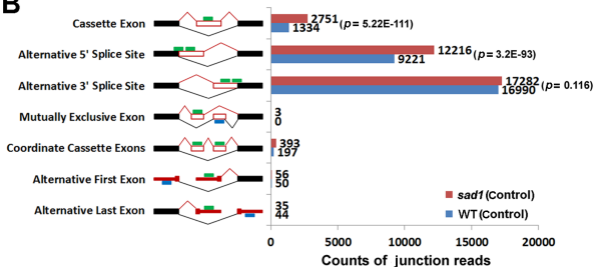

Supplement: Additional file 9 — Comparison of global AS between the wild type and sad1 under the control conditions. (A) The counts of each type of AS events in the wild type and sad1. The green/blue bars represent forward and reverse sequencing reads. (B) The total counts of the splice junction reads from each type of AS in the wild type and sad1. The P values were calculated by Fisher’s exact test comparing the junction read counts and the uniquely mapped reads between the wild type and sad1. [file gb-2014-15-1-r1-S9.pdf]

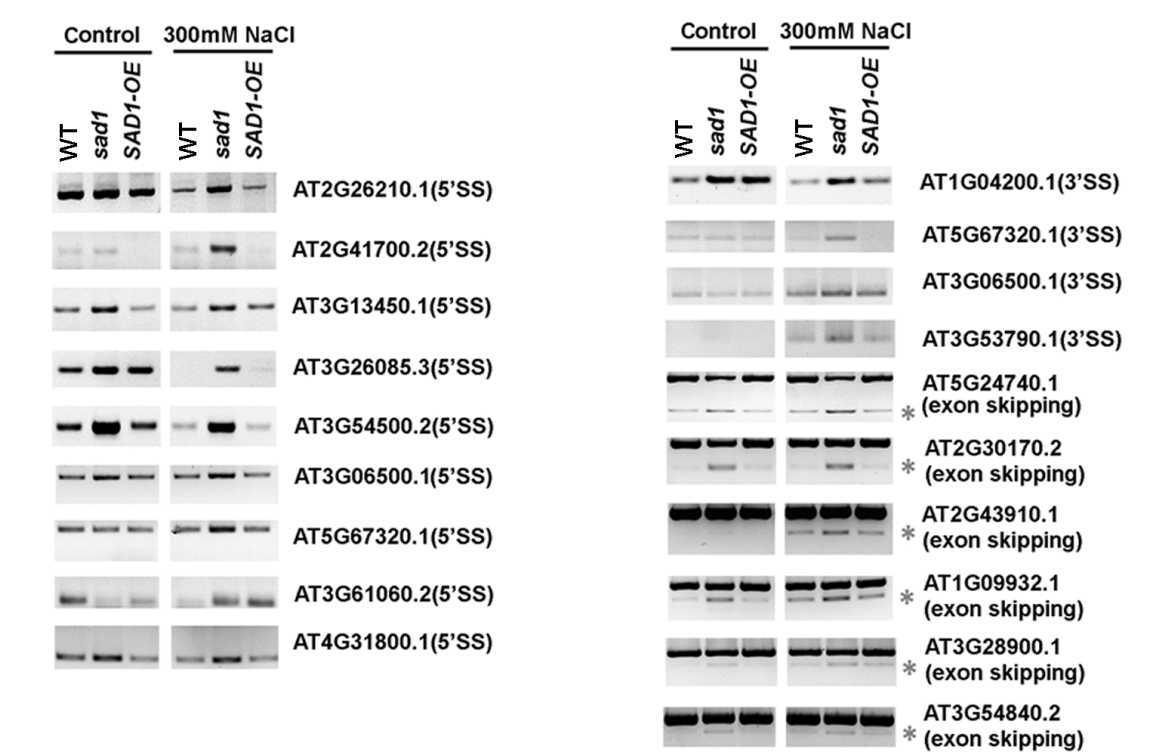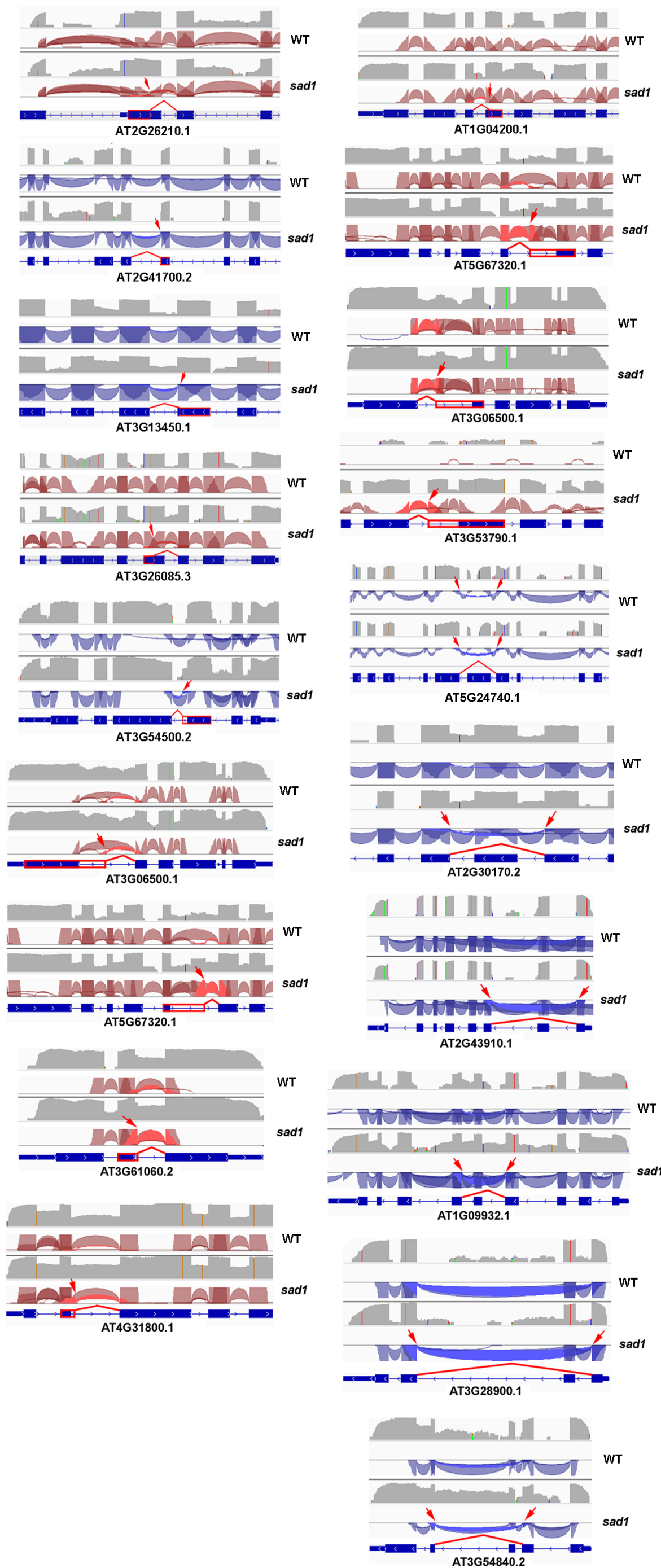

Supplement: Additional file 16 — Validation of the AS events in 19 genes by RT-PCR and corresponding IGV visualization. These 19 events include 9 alternative 5′SSs, 4 alternative 3′SSs and 6 exon-skipping events. For the validation of alternative 5′SSs and 3′SSs, there was only one band that represents the alternative-splice isoform, which was obviously detected in sad1 mutants, but not or only weakly detected in the wild type and SAD1-OE. For exon-skipping events, the alternatively spliced forms were marked with grey asterisks (*). For IGV visualization, alternative splicing sites were marked by red arrows and highlighted by red arcs. [file gb-2014-15-1-r1-S16.pdf]

**A***sad1* (Control)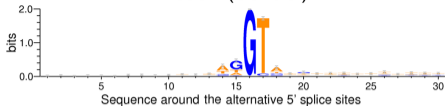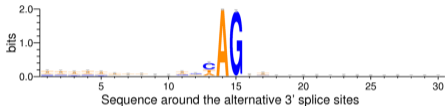**B***sad1* (Control)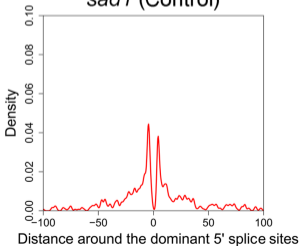

Supplement: Additional file 17 — The characters of activated alternative 5′SSs in sad1 under the control conditions. (A) The sequences around the alternative 5′SSs that were over-represented in the mutant were shown by Weblogo. (B) Distribution of activated alternative 5′SS around the dominant ones. These alternative 5′SSs were enriched in the downstream or upstream 10 bp region of the dominant 5′SSs (position 0 on the x- axis). [file gb-2014-15-1-r1-S17.pdf]

exon(Control)

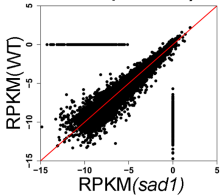

intron(Control)

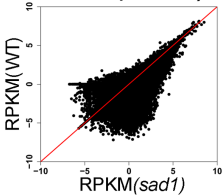

Supplement: Additional file 18 — Comparison of intron retention between the wild-type and sad1 plants under the control conditions. The RPKM values for the exons and introns were plotted. The expression of introns, but not exons, in the sad1 mutant showed a global up-regulation. [file gb-2014-15-1-r1-S18.pdf]

300 mM NaCl

WT

*sad1*

*SAD1-OE*

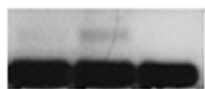

\*

AT1G20440(COR47)

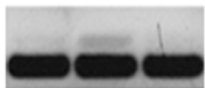

\*

AT1G76180(ERD14)

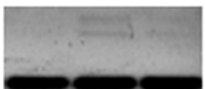

\*

AT3G19290(ABF4)

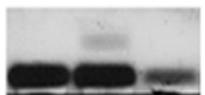

\*

AT4G39090(RD19)

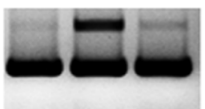

\*

AT5G62470(MYB96)

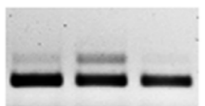

\*

AT1G27760(SAT32)

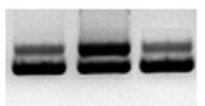

\*

AT1G68050(FKF1)

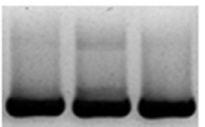

\*

AT4G26840(SUMO1)

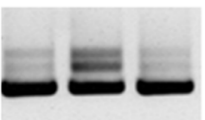

\*

AT3G55610(PSCS2)

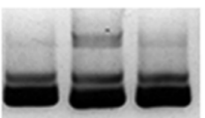

\*

AT2G40340(DREB2C)

Supplement: Additional file 19 — Validation of the intron retention in selected genes by RT-PCR using the intron-flanking primers. The intron retained splicing variants were marked by grey asterisks (*). [file gb-2014-15-1-r1-S19.pdf]

**NaCl**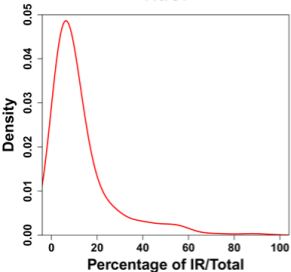**Control**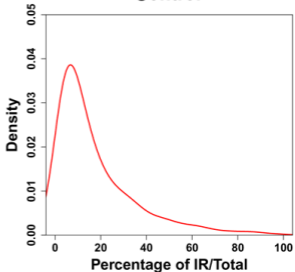

Supplement: Additional file 22 — Distribution of the proportions of intron-retained transcripts to the total transcripts for each gene with intron-retention in the sad1 mutants. The percentage is calculated by dividing the RPKM value of the retained intron by the RPKM value of the two-flanking exons. [file gb-2014-15-1-r1-S22.pdf]

# *sad1* (Control)

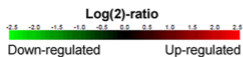

Bacteria  
infection

ABA

Cold

Drought

Salt

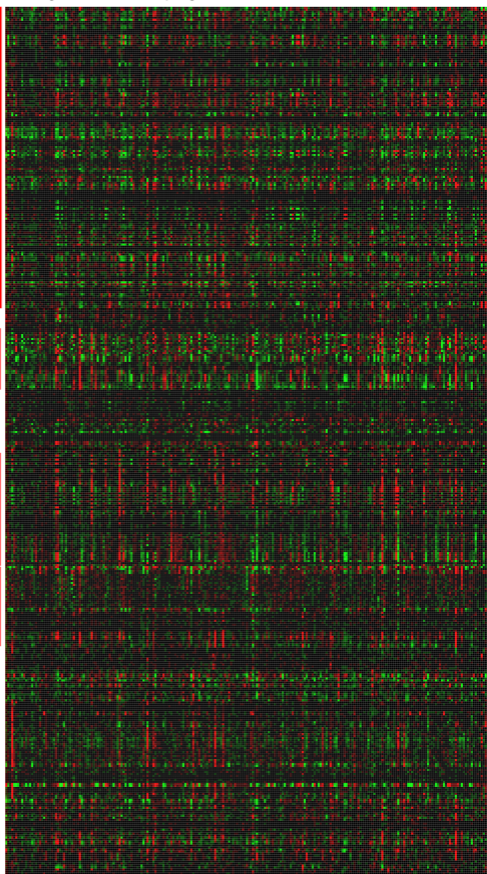

Supplement: Additional file 28 — A heatmap was generated by mapping the genes enriched at the response-to-abiotic-stress pathways to the microarray database at Genevestigator. The heatmap indicates that genes with abnormal splicing in the sad1 control treatment are not specifically related to the response to salt and ABA stress, but rather associated with random responses to various environmental stresses. [file gb-2014-15-1-r1-S28.pdf]

# Color Key

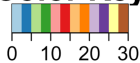

**Control**

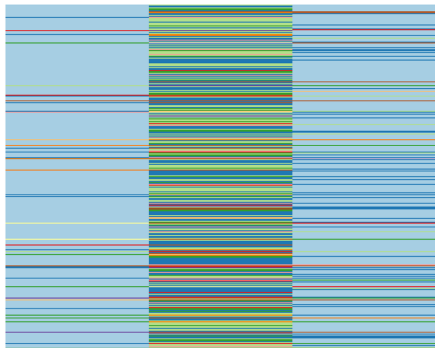

**WT**

***sad1***

***SAD1-OE***

Supplement: Additional file 30 — Profiling the normalized (by total uniquely mapped reads) read coverage of the splice junctions that were over-represented in the sad1 mutant relative to the wild type and SAD1-OE under the control conditions. The profiles indicate that the AS patterns in sad1 were completely or largely restored by overexpressing SAD1. [file gb-2014-15-1-r1-S30.pdf]

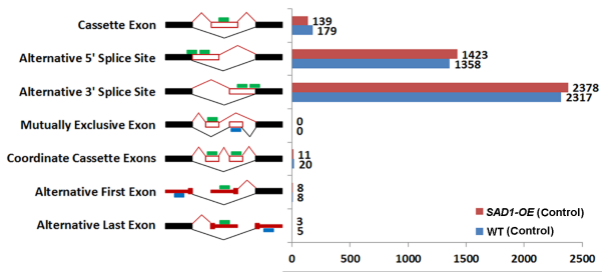

Supplement: Additional file 31 — Number of each type of AS event in the wild type and SAD1-OE under the control conditions. Note that the numbers of alternative 5′SSs, alternative 3′SSs and exon-skipping events in SAD1-OE are close to those in the wild type. The green/blue bars represent forward and reverse sequencing reads. [file gb-2014-15-1-r1-S31.pdf]

# Color Key

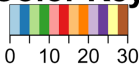

**Control**

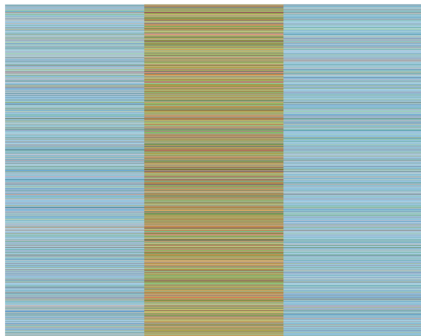

**WT**

***sad1***

***SAD1-OE***

Supplement: Additional file 33 — Profiling the normalized (by total uniquely mapped reads) read coverage of the introns that were over-represented in the sad1 mutant relative to the wild type and SAD1-OE under the control conditions. The profiles indicate that the intron retention events in sad1 were completely or largely restored by overexpressing SAD1. [file gb-2014-15-1-r1-S33.pdf]

**Total transcripts  
(NaCl)**

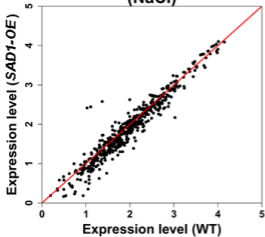

**Functional transcripts  
(NaCl)**

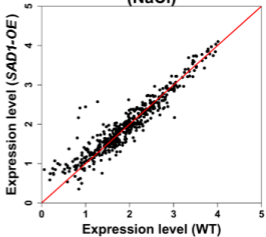

Supplement: Additional file 35 — Comparison of the total transcripts and functional transcripts (without retained intron) between the NaCl-treated wild-type and SAD1-OE plants. The reads number (log10) for the total transcripts and functional transcripts were plotted between the wild type and SAD1-OE. The functional transcripts tended to be up-regulated in the NaCl-treated SAD1-OE plants. [file gb-2014-15-1-r1-S35.pdf]

## Root growth under different NaCl concentration

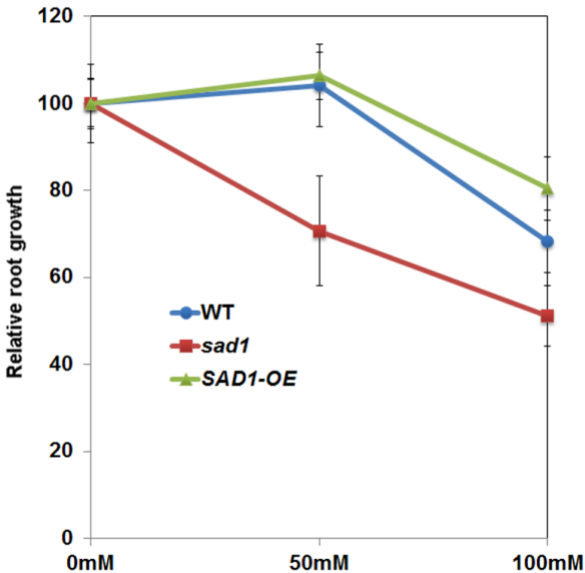

Supplement: Additional file 38 — Relative root length of the wild-type, sad1 and SAD1-OE seedlings after four days growth on 0, 50 or 100 mM NaCl. Data are means and standard errors from about 15 seedlings. One-week-old seedlings grown on ½ MS medium plates were transferred to ½ MS medium plates supplemented with the indicated concentrations of NaCl and allowed to grow for four days before measuring the root length. [file gb-2014-15-1-r1-S38.pdf]

# Control

WT

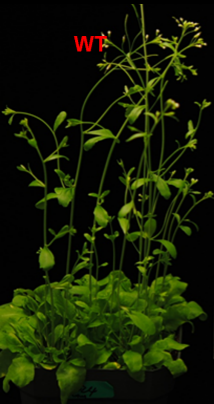

*sad1*

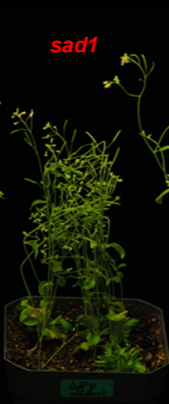

*SAD1-OE*

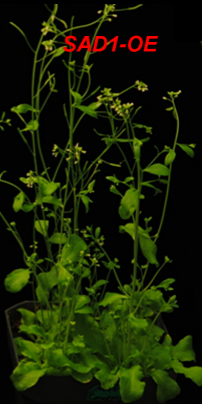

Supplement: Additional file 39 — Morphology of 28-day-old wild-type, sad1 and transgenic (SAD1-OE) plants grown under normal conditions (without NaCl treatments). [file gb-2014-15-1-r1-S39.pdf]

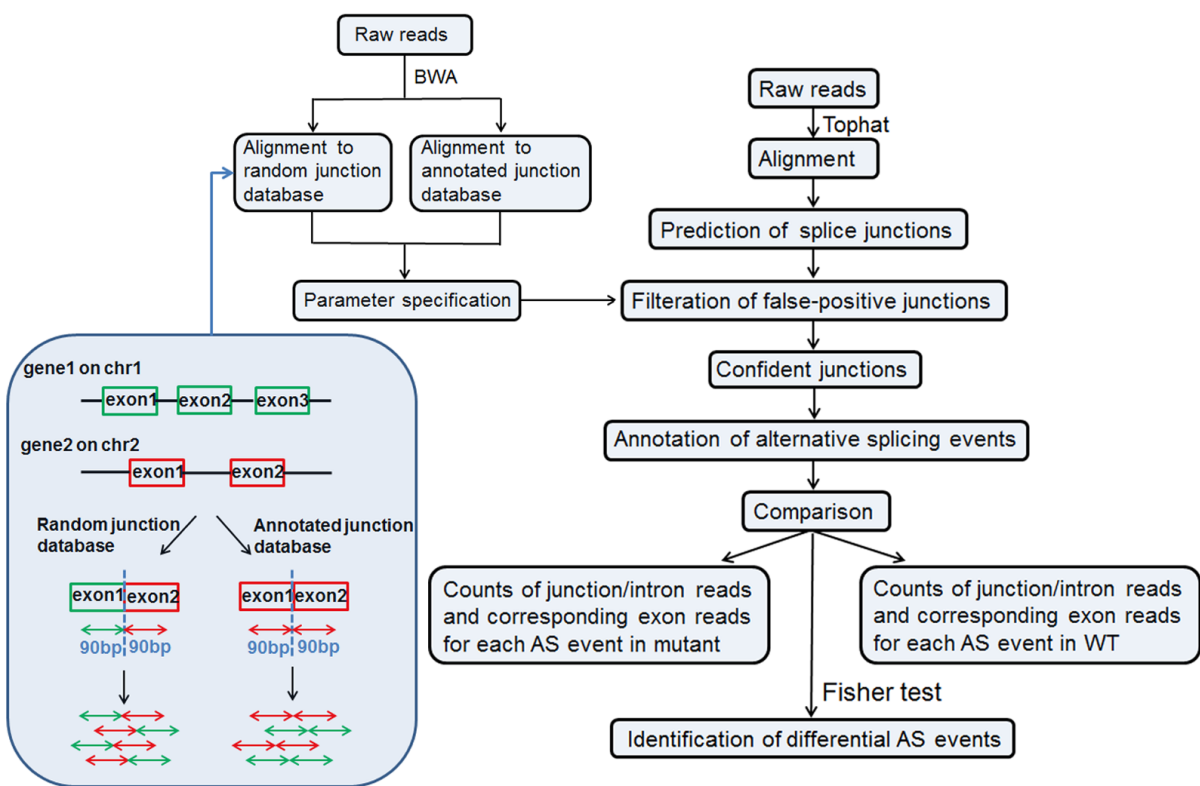

Supplement: Additional file 42 — The pipeline of RNA-seq data analysis in this study. The pipeline involves five steps: read alignment and junction prediction, the filter of false positive junctions, annotation of AS events, global comparison of AS and the identification of differential AS events. To estimate thresholds for filtering false positive junctions, two datasets of random and annotated splice junctions were created. The random splice junctions dataset was created by joining each annotated 5′ donor sequence (90/74 bp from 5′SSs) and the annotated 3′ donor sequence (90/74 bp from 3′SSs) located on a different chromosome. The annotated splice junctions dataset was created by joining each annotated 5′ donor sequence (90/74 bp from 5′SSs) and the annotated 3′ donor sequence (90/74 bp from 3′SSs) in order based on the gene annotation. [file gb-2014-15-1-r1-S42.pdf]
