# Supplementary material for: Dynamic regulation of genome-wide pre-mRNA splicing and stress tolerance by the Sm-like protein LSm5 in Arabidopsis
Source: Genome Biol. 2014 Jan 7;15(1):R1. doi: 10.1186/gb-2014-15-1-r1 (PMC4053965; doi:10.1186/gb-2014-15-1-r1)
Supplement: Additional file 7 — The features of false positive (random) and annotated junctions. (A) The density of the overhang size of false positive and annotated junctions. Most of false positive junctions show shorter overhang sizes, while the annotated junctions have larger overhang sizes. (B) The density of junction read coverage of false positives and annotated junctions. More than half of false positive junctions have only one read spanning the junction, while the annotated junctions have higher reads coverage. (C) Distinguishing true junctions from false positive alignments. To reduce the number of false positive junctions, as determined by randomly generated junctions, we required that the overhang size must be more than 20 bp (>20 bp) and at least two reads (>1 read) span the junctions. Using both criteria, the false positive junctions sharply reduced to very low levels (close to zero). By contrast, the annotated junctions show no obvious decrease. [file gb-2014-15-1-r1-S7.pdf]

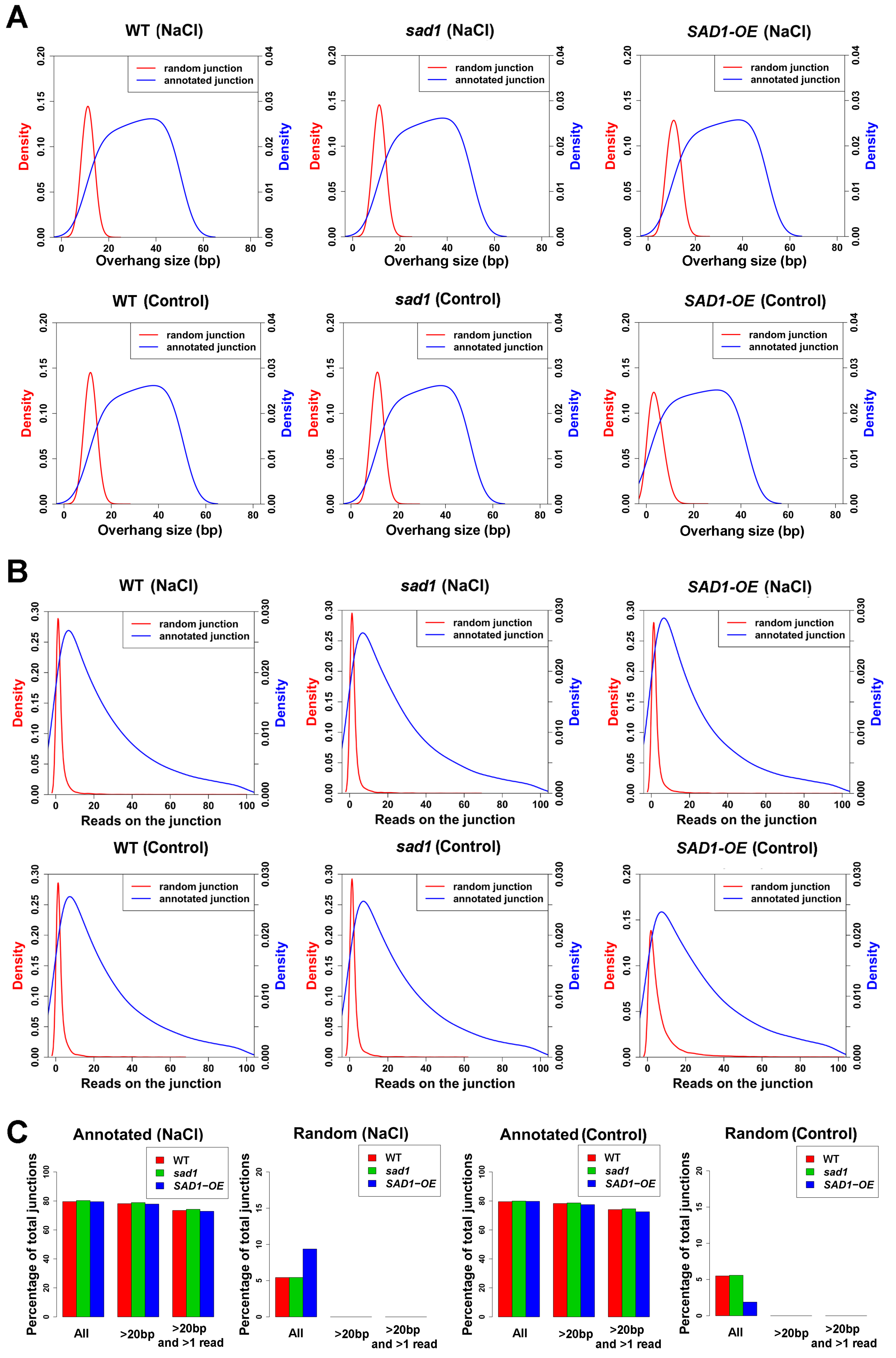
A
WT (NaCl)
sad1 (NaCl)
SAD1-OE (NaCl)
WT (Control)
sad1 (Control)
SAD1-OE (Control)
random junction
annotated junction
Density
Density
Overhang size (bp)
0.00 0.05 0.10 0.15 0.20
0.00 0.01 0.02 0.03 0.04
0 20 40 60 80
B
WT (NaCl)
sad1 (NaCl)
SAD1-OE (NaCl)
WT (Control)
sad1 (Control)
SAD1-OE (Control)
random junction
annotated junction
Density
Density
Reads on the junction
0.00 0.05 0.10 0.15 0.20 0.25 0.30
0.000 0.010 0.020 0.030
0 20 40 60 80 100
C
Annotated (NaCl)
Random (NaCl)
Annotated (Control)
Random (Control)
WT
sad1
SAD1−OE
Percentage of total junctions
0 20 40 60 80 100
0 5 10 15 20
All
>20bp
>20bp and >1 read
