# Supplementary material for: Dynamic regulation of genome-wide pre-mRNA splicing and stress tolerance by the Sm-like protein LSm5 in Arabidopsis
Source: Genome Biol. 2014 Jan 7;15(1):R1. doi: 10.1186/gb-2014-15-1-r1 (PMC4053965; doi:10.1186/gb-2014-15-1-r1)
Supplement: Additional file 23 — Comparison of the total transcripts and functional transcripts (without introns) between the wild type and sad1. The relative expression of total transcripts was measured as the read number of the two exons flanking the retained intron, and the relative expression of functional transcripts was calculated by deducting the expression of the retained intron (measured by the read number of the retained intron) from the expression of the total transcripts. The expression levels of the total transcripts did not show obvious change between the wild types and sad1, but the functional transcripts tended to be down-regulated in the control and NaCl-treated sad1 mutants. [file gb-2014-15-1-r1-S23.pdf]

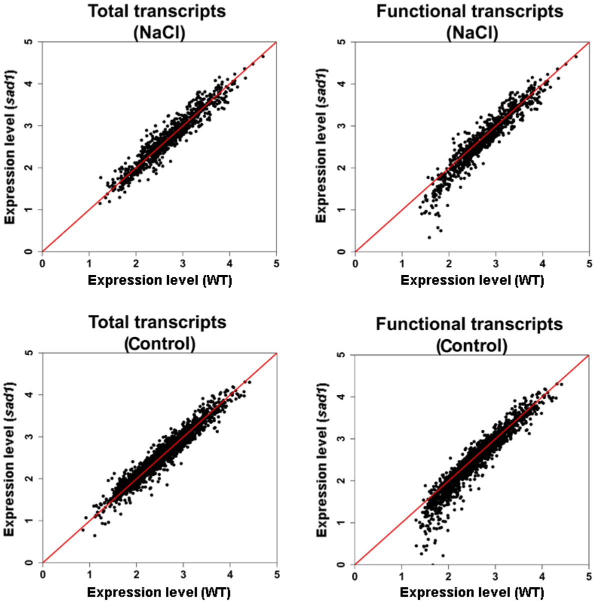
Total transcripts
(NaCl)
Functional transcripts
(NaCl)
Total transcripts
(Control)
Functional transcripts
(Control)
Expression level (sad1)
Expression level (WT)
0
1
2
3
4
5
