# Supplementary material for: Dynamic regulation of genome-wide pre-mRNA splicing and stress tolerance by the Sm-like protein LSm5 in Arabidopsis
Source: Genome Biol. 2014 Jan 7;15(1):R1. doi: 10.1186/gb-2014-15-1-r1 (PMC4053965; doi:10.1186/gb-2014-15-1-r1)
Supplement: Additional file 27 — A two-dimension view of the functional annotations of genes with abnormal splicing in sad1 under the control conditions. The functional classification of genes was done by using the DAVID software. The top 50 functional annotations ordered by the enrichment scores were selected for the two-dimensional view, which indicates that genes with abnormal splicing were strikingly enriched in the response-to-abiotic-stress category. [file gb-2014-15-1-r1-S27.pdf]

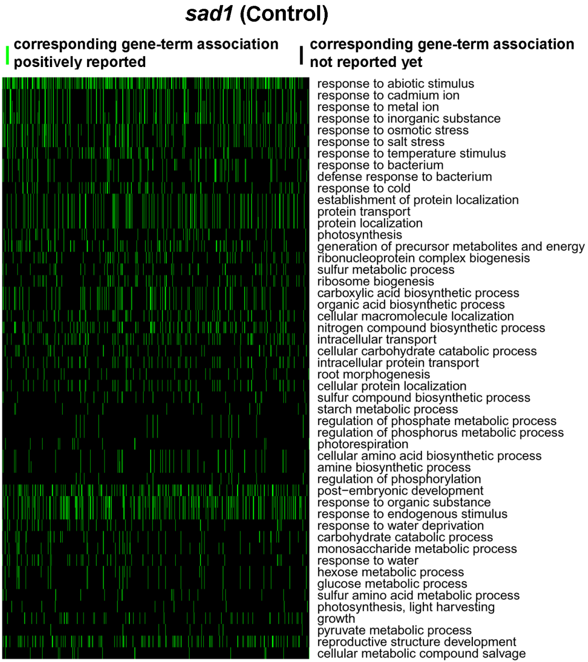
sad1 (Control)
corresponding gene-term association positively reported
corresponding gene-term association not reported yet
response to abiotic stimulus
response to cadmium ion
response to metal ion
response to inorganic substance
response to osmotic stress
response to salt stress
response to temperature stimulus
response to bacterium
defense response to bacterium
response to cold
establishment of protein localization
protein transport
protein localization
photosynthesis
generation of precursor metabolites and energy
ribonucleoprotein complex biogenesis
sulfur metabolic process
ribosome biogenesis
carboxylic acid biosynthetic process
organic acid biosynthetic process
cellular macromolecule localization
nitrogen compound biosynthetic process
intracellular transport
cellular carbohydrate catabolic process
intracellular protein transport
root morphogenesis
cellular protein localization
sulfur compound biosynthetic process
starch metabolic process
regulation of phosphate metabolic process
regulation of phosphorus metabolic process
photorespiration
cellular amino acid biosynthetic process
amine biosynthetic process
regulation of phosphorylation
post−embryonic development
response to organic substance
response to endogenous stimulus
response to water deprivation
carbohydrate catabolic process
monosaccharide metabolic process
response to water
hexose metabolic process
glucose metabolic process
sulfur amino acid metabolic process
photosynthesis, light harvesting
growth
pyruvate metabolic process
reproductive structure development
cellular metabolic compound salvage
