# Supplementary material for: Dynamic regulation of genome-wide pre-mRNA splicing and stress tolerance by the Sm-like protein LSm5 in Arabidopsis
Source: Genome Biol. 2014 Jan 7;15(1):R1. doi: 10.1186/gb-2014-15-1-r1 (PMC4053965; doi:10.1186/gb-2014-15-1-r1)

sad1 (Control)

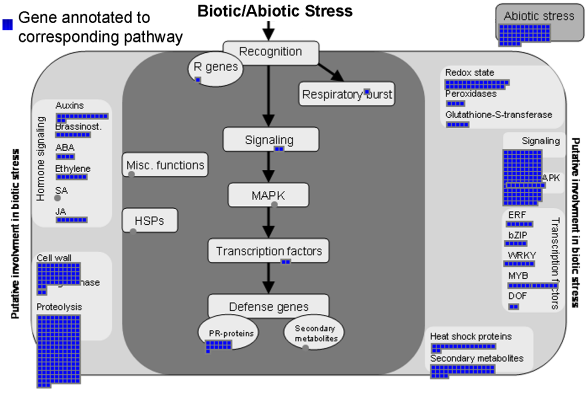

Supplement: Additional file 29 — A network generated by Mapman indicates that genes with aberrant splicing in the sad1 control treatment are involved in various stress response pathways, including hormone-signaling pathways, MAPK-signaling pathways and transcription regulation. [file gb-2014-15-1-r1-S29.pdf]
