# Supplementary material for: Dynamic regulation of genome-wide pre-mRNA splicing and stress tolerance by the Sm-like protein LSm5 in Arabidopsis
Source: Genome Biol. 2014 Jan 7;15(1):R1. doi: 10.1186/gb-2014-15-1-r1 (PMC4053965; doi:10.1186/gb-2014-15-1-r1)
Supplement: Additional file 37 — A network generated by Mapman indicates that genes with increased splicing efficiency in SAD1-OE are involved in various stress response pathways, including hormone-signaling pathways, MAPK-signaling pathways and transcription regulation. [file gb-2014-15-1-r1-S37.pdf]

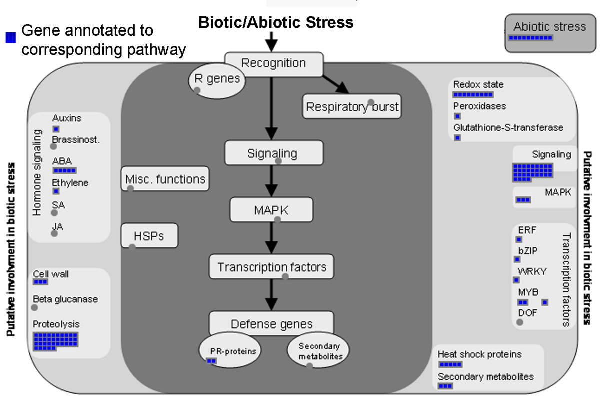

Gene annotated to corresponding pathway
Biotic/Abiotic Stress
Abiotic stress
Recognition
R genes
Respiratory burst
Signaling
MAPK
Transcription factors
Defense genes
PR-proteins
Secondary metabolites
Misc. functions
HSPs
Homone signaling
Auxins
Brassinost.
ABA
Ethylene
SA
JA
Cell wall
Beta glucanase
Proteolysis
Redox state
Peroxidases
Glutathione-S-transferase
Signaling
MAPK
Transcription factors
ERF
bZIP
WRKY
MYB
DOF
Heat shock proteins
Secondary metabolites
Putative involvment in biotic stress
Putative involvment in biotic stress
